# The accumulation of miR-125b-5p is indispensable for efficient erythroblast enucleation

**DOI:** 10.1038/s41419-022-05331-5

**Published:** 2022-10-21

**Authors:** Fang Fang, Lei Xu, Liqing Liang, Mingyi Qu, Hailei Yao, Wen Yue, Lin Chen, Dongli Chen, Zeng Fan, Lijuan He, Xue Nan, Huanhuan Zhang, Xiaoyan Xie, Xuetao Pei

**Affiliations:** 1 Stem Cell and Regenerative Medicine Lab, Beijing Institute of Radiation Medicine, Beijing, 100850 China; 2 South China Research Center for Stem Cell & Regenerative Medicine, Guangzhou, 510005 China; 3 Department of State-owned Assents and Laboratory Management, Capital Medical University, Beijing, 100069 China; 4 Institute of Health Service and Transfusion Medicine, Beijing, 100850 China; 5 Second Department of Oncology, First Affiliated Hospital of PLA General Hospital, Beijing, 100048 China

**Keywords:** Haematopoietic stem cells, miRNAs

## Abstract

Erythroblast enucleation is a precisely regulated but not clearly understood process. Polycythemia shows pathological erythroblast enucleation, and we discovered a low miR-125b-5p level in terminal erythroblasts of patients with polycythemia vera (PV) compared to those of healthy controls. Exogenous upregulation of miR-125b-5p levels restored the enucleation rate to normal levels. Direct downregulation of miR-125b-5p in mouse erythroblasts simulated the enucleation issue found in patients with PV, and miR-125b-5p accumulation was found in enucleating erythroblasts, collectively suggesting the importance of miR-125b-5p accumulation for erythroblast enucleation. To elucidate the role of miR-125b-5p in enucleation, gain- and loss-of-function studies were performed. Overexpression of miR-125b-5p improved the enucleation of erythroleukemia cells and primary erythroblasts. Infused erythroblasts with higher levels of miR-125b-5p also exhibited accelerated enucleation. In contrast, miR-125b-5p inhibitors significantly suppressed erythrocyte enucleation. Intracellular imaging revealed that in addition to cytoskeletal assembly and nuclear condensation, miR-125b-5p overexpression resulted in mitochondrial reduction and depolarization. Real-time PCR, western blot analysis, luciferase reporter assays, small molecule inhibitor supplementation and gene rescue assays revealed that Bcl-2, as a direct target of miR-125b-5p, was one of the key mediators of miR-125b-5p during enucleation. Following suppression of Bcl-2, the activation of caspase-3 and subsequent activation of ROCK-1 resulted in cytoskeletal rearrangement and enucleation. In conclusion, this study is the first to reveal the pivotal role of miR-125b-5p in erythroblast enucleation.

## Introduction

Erythroblast enucleation, one of the steps of erythroid terminal maturation, is a critical step of erythropoiesis, and this process is intricately regulated. As a consequence, nucleated RBCs (NRBCs) are rarely found in human circulation. However, little is known regarding how mammalian erythrocytes extrude their nucleus [1]. Several chromatin remodeling enzymes, cytoskeletal regulators and miRNAs have been discovered to play roles in erythroblast enucleation [2–6]. Among these regulators, miRNAs are involved in multiple physiological and pathological processes, and the same miRNA may function differently according to the intracellular context.

miR-125b-5p, a regulator of hematopoiesis, is dysregulated in blood malignancies and affects the self-renewal, proliferation and differentiation of various types of blood cells, including hematopoietic stem cells (HSCs), common myeloid progenitors, megakaryocytic/erythroid progenitors and megakaryocytic progenitors [7–9]. In Down’s syndrome (DS), trisomy 21 results in overexpression of miR-125b-5p, which is one of the key causes of DS-related acute megakaryoblastic leukemia, and miR-125b-5p is a positive regulator of megakaryopoiesis [10]. Based on the hematopoiesis differentiation tree, megakaryocytes have the closest relationship with erythrocytes among different types of blood cells [11]. Therefore, it is unsurprising that infants with DS also have a high risk of developing polycythemia [12, 13]. Interestingly, true adult polycythemia vera (PV) is also commonly associated with the upregulation of miR-125b-5p [14].

PV is a condition that occurs when bone marrow (BM) abnormally and excessively produces RBCs. The abnormal increase in RBCs may cause blood to thicken and clot. Moreover, 95% of patients with PV have the JAK2V617F mutation, and NRBCs are often observed in peripheral blood (PB) smears from these patients [15, 16]. This finding suggests a causal relationship between miR-125b-5p dysregulation and abnormal erythroblast maturation. To elucidate the role of miR-125b-5p in erythroblast enucleation, we examined the endogenous expression of miR-125b-5p during erythropoiesis of hematopoietic stem/progenitor cells from both healthy controls and patients with PV. The effect of miR-125b-5p gene modulation on erythroblast enucleation was further examined, and the downstream signal of miR-125b-5p during enucleation was also investigated.

## Results

### Abnormal miR-125b-5p expression is responsible for increased NRBCs in patients with PV

**Fig. 1:**
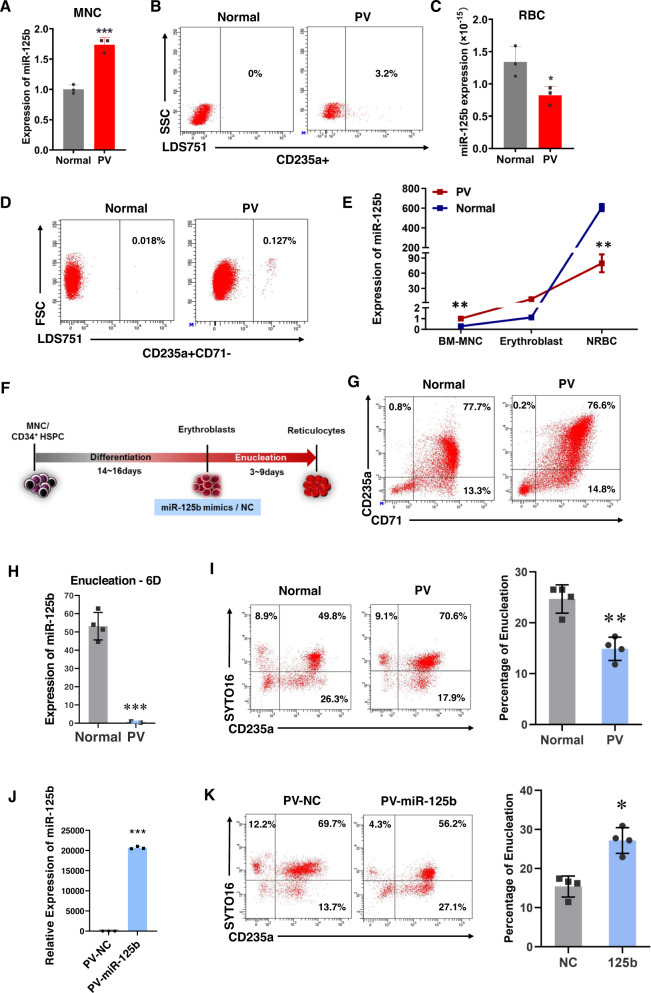
Abnormal expression of miR-125b-5p and its correlation with increased nucleated RBCs in patients with polycythemia vera. **A** Quantitative RT-PCR shows endogenous miR-125b-5p expression in MNCs isolated from the PB of healthy controls and patients with PV (*n* = 3). Relative expression was normalized to U6 miRNA and is expressed as the mean ± SD (*p* = 0.0008). **B** Flow cytometry analysis shows the NRBC percentage in the PB of healthy controls or patients with PV. **C** qRT-PCR of miR-125b-5p expression from three independent experiments with healthy RBCs or RBCs from patients with PV. The results were normalized with cell number (*p* = 0.0309). **D** Representative images of the flow cytometry analysis of nucleated (LDS751^+^) CD235a^+^CD71^−^ erythrocytes (NRBCs) in the BM of healthy controls and patients with PV. **E** qRT-PCR using the indicated cells isolated from the BM of healthy controls and patients with PV shows the expression trend of miR-125b-5p during erythropoiesis (MNCs, erythroblasts and NRBCs; **p = 0.0058). **F** Schematic illustration representing the erythrocyte induction procedure and transfection with the miR-125b-5p mimic. **G** Representative images of flow cytometry analysis of CD71 and CD235a expression at erythroblast differentiation Day 14 on BM-MNCs from healthy controls and patients with PV. On enucleation Day 6, qRT-PCR (*p* = 0.0007) shows miR-125b-5p expression (**H**), and representative images show the percentage of enucleated erythroblasts (CD235a^+^/SYTO 16^−^) from healthy controls and patients with PV (**I**) The right panel of (**I**) shows the statistical analysis of data from four independent flow cytometry analyses, and the bar graph represents the mean ± SD (*p* = 0.0016). **J** qRT-PCR shows the overexpression of miR-125b-5p (*p* = 3.09489E-06) and representative images of flow cytometry analysis (**K**) illustrate the enucleation status of PV erythroblasts after treatment with miR-125b-5p mimic or control miRNA. The right panel of (**K**) shows the statistical analysis of data from four independent flow cytometry analyses, and the bar graph represents the mean ± SD (*p* = 0.0032; *p < 0.05, **p < 0.01, and ***p < 0.001).

To elucidate the potential role of miR-125b-5p in erythroid terminal maturation in patients with PV, we first compared the expression levels of miR-125b-5p in mononuclear cells (MNCs) and RBCs isolated from patients with PV or healthy controls. The expression of miR-125b-5p in PV-MNCs was higher than that in normal MNCs (Fig. 1A) as previously reported [10]. In addition, up to 3.2% of NRBCs in PB from patients with PV were detected by flow cytometry, whereas NRBCs in healthy controls were barely detected (Fig. 1B). Interestingly, the expression of miR-125b-5p was markedly suppressed in mature RBCs from patients with PV (Fig. 1C). These observations suggested a correlation between low miR-125b-5p levels and erythroblast enucleation issues.

The level of miR-125b-5p was also examined in erythrocytes one step before enucleation (late erythroblasts) in CD71^−^ (early stage erythroid marker), CD235a^+^ (late-stage marker) and LDS751^+^ cells. CD235^+^CD71^−^LDS751^+^ cells (NRBCs) were sorted from the BM biopsies of patients with PV because these cells are not mature enough to be released into PB. Similar to PB, the percentage of NRBCs in the BM of patients with PV was higher than that in healthy controls (Fig. 1D). Interestingly, a phased comparison of miR-125b-5p expression revealed that although PV miR-125b-5p remained higher than healthy miR-125b-5p in CD235^+^CD71^+^LDS751^+^ erythroblasts sorted from BM, the expression pattern was markedly inverted by the NRBC stage (Fig. 1E). The same trend was present in mature RBCs as shown in Fig. 1C. Accordingly, we hypothesized that a certain level of miR-125b-5p upregulation is required for terminal erythroblast enucleation.

To confirm that lower miR-125b-5p expression impairs erythroblast enucleation in patients with PV, we examined hematopoietic stem/progenitor cell erythropoiesis in PV patients in vitro. A stepwise cell culture protocol was developed permitting human cord blood (hCB)-isolated CD34^+^ cells or MNCs to differentiate and enucleate toward erythrocytes in vitro (Fig. S1A). By Day 14 of culture, the percentage of CD235a^+^CD71^+^ cells reached 85% ± 10% (Fig. S1B and C), which suggested that the cells were primary basophilic normoblasts. The cells were then transferred to enucleation medium for an additional 4–6 days, and the output SYTO16^-^CD235a^+^ cells were considered as enucleated erythrocytes (Fig. S1B) [17, 18]. Under in vitro induction conditions, the erythroid differentiation pattern of PV patient BM-MNCs showed no difference compared to healthy controls as indicated by the percentages of CD71^+^ and CD235a^+^ cells (Fig. 1G). But erythroblasts derived from PV MNCs performed better in CD71^high^/CD235a^high^ population development, which matched the consequences of the JAK2 V617F mutation (Fig. S2) and higher erythroid progenitor expansion in these patients [19, 20]. However, similar to the examination with primary counterparts, the PV group exhibited a low miR-125b-5p level after 7 days of second stage induction compared to the healthy control group (Fig. 1H). As expected, the enucleation efficiency (percentage of CD235a^+^SYTO16^-^ and CD71^+^SYTO16^-^ cells) of BM-MNC-derived erythroblasts in the PV group was significantly lower than that in the control group (Fig. 1I). In addition, exogenous miR-125b-5p overexpression with miRNA mimics (Fig. 1J) functionally restored the enucleation efficiency to a normal level (Fig. 1K). These findings indicated a potential correlation between decreased miR-125b-5p levels and elevated NRBCs.

### Erythroblast-specific miR-125b-5p knockdown impairs enucleation

**Fig. 2:**
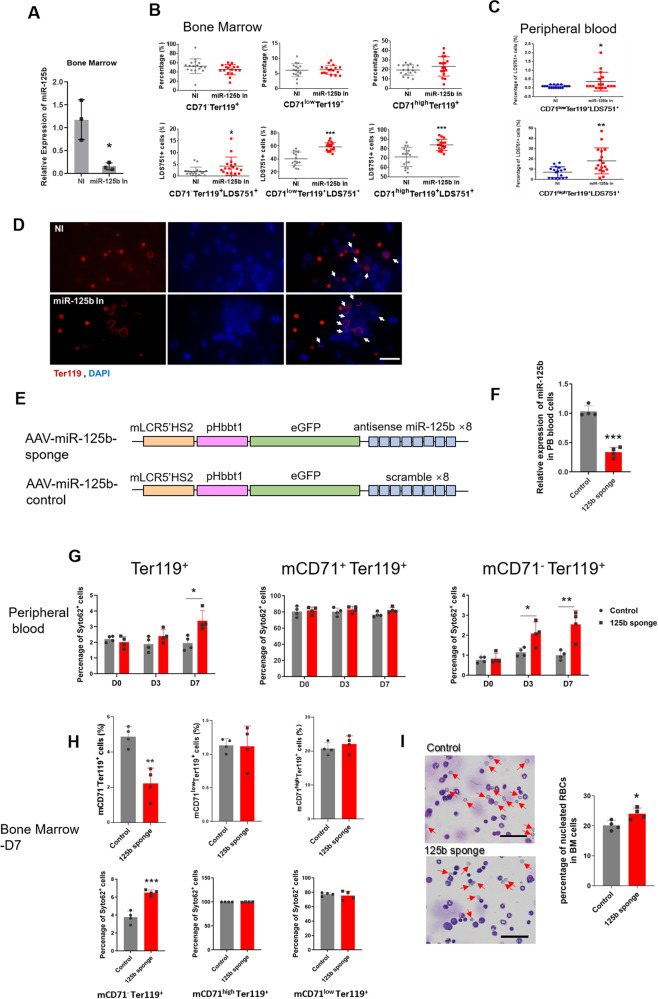
miR-125b-5p inhibition impairs the terminal enucleation of erythroblasts in vivo. **A** After intra-BM injection of miR-125b-5p inhibitors in ICR mice, qRT-PCR demonstrated the decreased expression of miR-125b-5p in BM (*p* = 0.0162). U6 was the loading control, and the results are expressed as the mean ± SD (*n* = 3). **B**, **C** Comparison of the erythroblast differentiation and enucleation rate between the miR-125b-5p inhibitor injection group and the control group (17 mice per group) on injection Day 3. Statistical analysis of the percentage of CD71^−^/TER-119^+^, CD71^low^/TER-119^+^ and CD71^high^/TER-119^+^ erythroblasts (**B**, upper panel, no statistically significant difference) and nucleated cells (LDS751^+^) in CD71^−^/TER-119^+^, CD71^low^/TER-119^+^ and CD71^high^/TER-119^+^ erythroblasts harvested from BM (**B**, lower panel, *p* = 0.0391, *p* = 6.91972E-07 and *p* = 7.11841E-05) and nucleated cells from PB (**C**, *p* = 0.0431 and *p* = 0.0023). Data are expressed as the mean ± SD (*p < 0.05, **rp < 0.01, and ***p < 0.001). **D** Representative images of immunofluorescence staining show cells derived from BM with or without miR-125b-5p inhibitor treatment. miR-125b-5p inhibitor treatment resulted in increased NRBCs in BM. Red indicates Ter119 staining, and blue indicates DAPI nuclear staining. The arrows indicate Ter119^+^ cells with nuclei (i.e., NRBCs). Scale bars = 25 μm. **E** Schematics illustrating AAV vectors that carried miR-125b-5p sponge or control. mLCR5'HS2 represents the enhancer of mouse beta globin and pHbbt1 represents the promoter of mouse beta globin. **F** Seven days after tail vein injection of the miR-125b-5p sponge in BALB/c mice, qRT-PCR demonstrated the decreased expression of miR-125b-5p in PB (*p* = 0.0001). U6 was the loading control, and the results are expressed as the mean ± SD (*n* = 4). **G** Comparison of the erythroblast enucleation rate between the miR-125b-5p sponge injection group and the control group (4 mice per group) on injection Days 0, 3 and 7. Statistical analysis of the ratio of nucleated cells (Syto62^+^) in whole red blood cells (Ter119^+^) (*p* = 0.0388 for Day 7) as well as TER119^+^/CD71^+^ and TER119^+^/CD71^−^ erythroblasts (*p* = 0.0353 and *p* = 0.0273 for Day 3 and Day 7 TER119^+^/CD71^−^ erythroblasts respectively) harvested from PB. Data are expressed as the mean ± SD (*p < 0.05). **H** Comparison of the erythroblast differentiation and enucleation rate between the miR-125b-5p sponge injection group and the control group (4 mice per group) from BM erythroid cells at Day 7 post injection. Upper panel is statistical analysis of erythroblast differentiation, significant difference was only detected in CD71^−^/TER119^+^ erythroblasts (*p* = 0.0022). Lower panel is statistical analysis of the ratio of nucleated cells (Syto62^ +^ ) in TER119^+^/CD71^high^, TER119^+^/CD71^low^ and TER119^+^/CD71^−^ erythroblasts harvested from BM (*p* = 0.0004 for TER119^+^/CD71^−^ erythroblasts). Data are expressed as the mean ± SD (***p < 0.001). **I** Left panel: Representative images of the bone marrow smear stained with Wright-Giemsa dye. Arrows indicate the enucleated cells. Scale bars = 50 μm. Right panel: Statistical analysis of the percentage of BM nucleated cells calculated from four random views. miR-125b-5p sponge treatment resulted in increased NRBCs in BM. (*p* = 0.0193) (*p < 0.05).

Because miR-125b-5p functions inconsistently in different developmental stages, two miR-125b-5p knockdown (KD) mouse models were generated to further address the relationship between miR-125b-5p suppression and enucleation disorder. In a hematopoietic common KD model, miR-125b-5p inhibitors (miR-125b In) were injected into the BM cavity of Institute of Cancer Research (ICR) model mice. The impact of miR-125b-5p KD on NRBC occurrence in BM and PB was estimated by flow cytometry in a short period postinjection. Considering that terminal erythroblast maturation presents as full induction of Ter119 and sequential downregulation of CD71, [21] we separated Ter119^+^ erythroblasts by CD71 expression and examined nucleated cells in Ter119^+^CD71^high^, Ter119^+^CD71^low^, and Ter119^+^CD71^−^ populations, which represent sequentially matured erythroblasts. Four days after inhibitor injections, miR-125b-5p expression was successfully downregulated in the examined tissues (Fig. 2A). Similar to the discovery in patients with PV, the downregulation of miR-125b-5p significantly increased the percentage of NRBCs in BM (Figs. 2B, D and S3) and PB (Fig. 2C) without altering the proportion of each erythroblast populations (Fig. 2B).

Erythroblast-specific miR-125b-5p KD was established using adenovirus-associated virus (AAV) tail vein injection. Briefly, beta globin enhancer- and promoter-carrying eGFP-miR-125b sponges (8-repeat antisense-miR-125b-5p) were inserted into the AAV vector, and 8-repeat scramble nucleotides were set as a control (Fig. 2E). As confirmed with Day 7 PB samples, the sponge significantly reduced miR-125b-5p expression (Fig. 2F). By Days 3 and 7 post- AAV injection, more NRBCs (SYTO62 positive) were found in the sponge group PB, especially in the Ter119^+^CD71^−^ populations (Fig. 2G). BM samples were examined 7 days post- AAV injection, and more NRBCs were detected in both the FCM and cell smears of the sponge group together with reduced Ter119^+^CD71^−^ final stage erythroblast populations (Figs. 2H and I). Similar to the finding in PB, a significant difference was only found in the Ter119^+^CD71^−^ populations. (Representative flow cytometry plots are shown in Fig. S4). Considering that active globin expression occurs in the late stage of erythropoiesis, [22] the miR-125b-5p sponge controlled by globin regulators showed no effect on BM HSC colony-forming ability (Fig. S5). Consistently, most of the hemogram indices remained intact except for higher MCHC and MCH after miR-125b-5p KD (Fig. S6). Impaired enucleation by the miR-125b-5p sponge might contribute to these changes.

### miR-125b-5p is essential for in vitro erythroblast enucleation

**Fig. 3:**
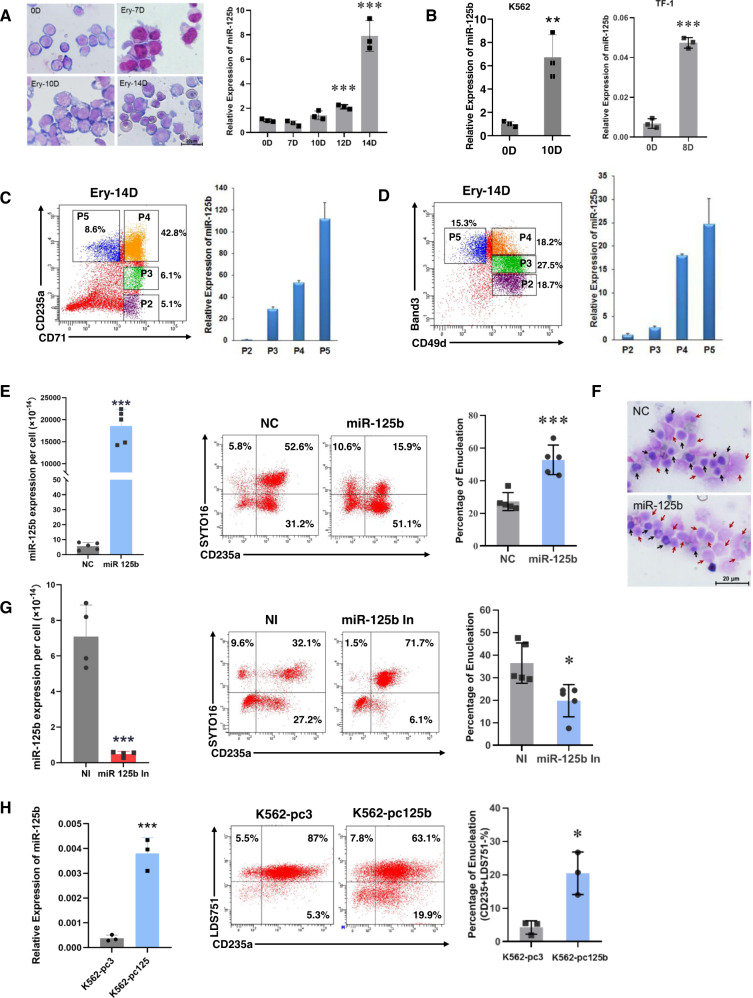
miR-125b-5p expression is positively correlated with erythroblast enucleation efficiency. **A** CD34^+^ hematopoietic stem/progenitor cells (HSPCs) were induced toward erythrocytes. Erythroid maturation was visualized by staining with May-Grünwald Giemsa on Days 0, 7, 10 and 14 (left panel). Scale bars = 20 μm. The expression of miR-125b-5p during erythropoiesis on Days 0, 7, 10, 12 and 14 was determined by qRT-PCR (right panel, *p* = 0.0008 for Day 12 and *p* = 0.0007 for Day 14). **B** miR-125b-5p was significantly upregulated in the erythroid enucleation induction of K562 (on Day 10) and TF-1 (on Day 8) cells (*p* = 0.0065 for K562 and *p* = 4.03263E-05 for TF-1). **C**, **D** miR-125b-5p expression in different erythroid developmental stages. In CD34^+^ HSPCs at erythroid induction day 14, erythroblasts were stained and sorted by CD71/CD235a (**C**) or CD49d (α4 integrin)/Band 3 (**D**) In the CD71/CD235a system (**C**), populations were gated as follows: P2, CD71^+^CD235a^-^; P3, CD71^+^CD235a^med^; P4, CD71^+^CD235a^+^; and P5, CD71^−^CD235a^+^ (representative cytospin images of the sorted populations are shown below). In a CD49d/Band 3 system (**D**), populations were gated as follows: P2, CD49d^+^Band 3^-^; P3, CD49d^+^Band 3^med^; P4, CD49d^+^Band 3^+^; and P5, CD49d^-^Band 3^+^. qRT-PCR analysis (right panel) demonstrated elevated miR-125b-5p levels from P2 to P5. U6 served as the miRNA expression control. **E** Exogenous upregulation of miR-125b-5p in hCB-MNC-derived erythroblasts enhanced the erythroblast enucleation rate. Overexpression of miR-125b-5p was analyzed by qRT-PCR (left panel, *p* = 7.82165E-06). Representative images of flow cytometry analysis show the enucleation efficiency (CD235^+^SYTO 16^-^, center panel). Statistical analysis of the enucleation rates from five independent experiments is expressed as the mean ± SD (right panel; *p* = 0.0006). **F** Representative images of the enucleation morphology on stained cytospins are shown. Red arrows indicate enucleated cells, and black arrows indicate nucleated cells. Scale bars = 20 μm. **G** Exogenous downregulation of miR-125b-5p in erythroblasts reduced their enucleation rate. Left panel shows miR-125b-5p levels after inhibitor transfection (*p* = 0.0012). Middle panel shows representative flow cytometry analysis of erythroblast enucleation. Right panel shows statistical enucleation rate (*p* = 0.0114). Statistical analysis was based on data from five independent experiments. **H** Stable miR-125b-5p overexpression improved K562 erythroid cell enucleation. The expression of miR-125b-5p was detected with qRT-PCR (left panel, *p* = 0.0008). Overexpression of miR-125b-5p by the pcDNA3.1 vector in K562 cells increased the percentage of CD235a^+^LDS751^−^ populations. Statistical analysis of three independent experiments is expressed as the mean ± SD (right panel) (*p* = 0.0135). In qRT-PCR analyses, U6 was the loading control in (**A**–**D**) and (**H**), while cell number was chosen as the loading control in (**E**) and (**G**), and the results are expressed as the mean ± SD (*n* = 3; *p < 0.05, **p < 0.01, and ***p < 0.001).

We used an in vitro erythrocyte induction and maturation system (Fig. S1 and Fig. 1F) to monitor the endogenous expression of miR-125b-5p. miR-125b-5p was gradually upregulated during primary cell erythropoiesis (Fig. 3A). For the erythroleukemia cell lines, [23, 24] significant upregulation of miR-125b-5p was also detected in K562 and TF-1 erythroid enucleating cells (Fig. 3B). The expression of miR-125b-5p was further examined by the developmental stage of erythroblasts. Around induction Days 14–16, there are commonly five distinct populations of erythroblasts as indicated by the CD235a/CD71 or Band3/CD49d double-staining patterns, [25, 26] and these populations are classified as P1-P5 according to the elevated maturation level. In the present study, the P1 populations that were CD71^low^CD235a^−^ or CD49d^low^Band3^−^ are not shown. Cytospins of the P2-P5 populations sorted by CD235a/CD71 demonstrated morphological changes during erythropoiesis. Most of the cells at this stage were nucleated, as measured by CD71/Syto16 and CD235a/Syto16 flow cytometry analysis (Fig. S7). The endogenous expression of miR-125b-5p steadily increased from P2 to P5 as shown by qRT-PCR (Fig. 3C, D), suggesting that elevated miR-125b-5p is required for successful maturation and enucleation.

We next conducted miR-125b-5p gain- and loss-of-function studies to further define the correlation between miR-125b-5p expression and enucleation. To avoid the delayed effect from early erythropoiesis with miR-125b-5p modification, basophilic erythroblasts at induction Day 14 were selected for miRNA transfection and the enucleation study. We transfected these hCB-MNC-derived basophilic erythroblasts with a miR-125b-5p mimic (miR-125b), miR-125b-5p inhibitor (miR-125b In) and corresponding controls (NC and NI, respectively). The transfection efficiency was measured by qRT-PCR (Fig. 3E left and 3 G left) and FACS with the aid of a FAM-stained miRNA mimic (Fig. S8). Five days after transfection, the enucleation efficiency was increased with miR-125b-5p overexpression (Fig. 3E). The impact of miR-125b-5p overexpression on enucleation was further confirmed by cytospin staining as more enucleated erythrocytes were observed in the images with higher miR-125b-5p levels (Fig. 3F). Correspondingly, miR-125b-5p suppression resulted in impaired enucleation (Fig. 3G). Using erythroleukemia cells, we generated a stable miR-125b-5p-overexpressing K562 cell line (K562-pc125b) for the miRNA function study. Overexpression of miR-125b-5p in K562 erythroid cells also significantly enhanced erythroblast enucleation as shown by the CD235^+^LDS751^−^ population percentage (Fig. 3H).

### miR-125b-5p overexpression promotes erythroblast enucleation in vivo

**Fig. 4:**
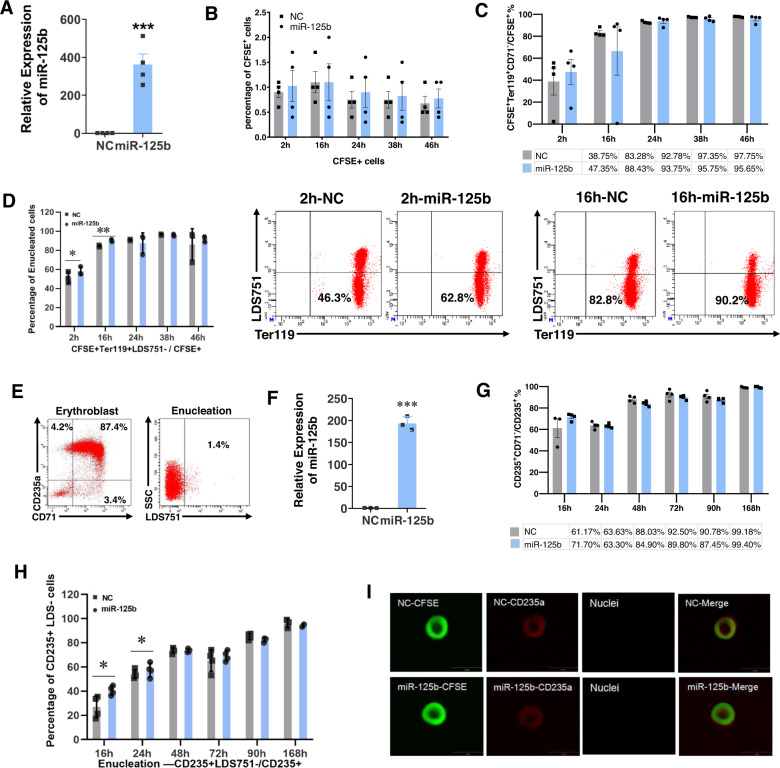
miR-125b-5p increases the enucleation of mFL- and hCB-MNC-derived erythroblasts in vivo. **A**–**D** miR-125b-5p increases mFL-derived erythroblast enucleation in vivo. **A** qRT-PCR shows the relative expression of miR-125b-5p after miRNA mimic transfection. U6 was the loading control, and the results are expressed as the mean ± SD (*n* = 4; *p* = 0.0006). **B** The kinetics of CFSE-labeled erythroblast retention in mouse PB after infusion. The results are expressed as the mean ± SD (*n* = 4). **C** The percentage of TER-119^+^CD71^−^ populations in CFSE^+^ erythroblasts was increased after cell infusion. **D** Compared to the control, miR-125b-5p overexpression promoted early stage enucleation of infused erythroblasts, especially at 2 and 16 h post infusion. Representative flow cytometry analysis shows the enucleation percentage at 2 and 16 h. Quantitative analysis of three independent experiments is shown as the mean ± SD (*p* = 0.044 and *p* = 0.003 for 2 and 16 h, respectively). **E–I** miR-125b-5p increased hCB-MNC-derived erythroblast enucleation in vivo. **E** Immediately before infusion, most hCB-MNC-derived erythroblasts were CD235a and CD71 double positive and nucleated (LDS751 positive). **F** qRT-PCR analysis of the relative expression of miR-125b-5p after miRNA mimic transfection. U6 was the loading control, and the results are expressed as the mean ± SD (*n* = 3; *p* = 2.90811E-05). **G** In recipient mouse PB, the percentage of CD71^−^ populations in CD235a^+^ human erythroblasts was increased after cell infusion. **H** Compared to the control, miR-125b-5p overexpression increased the early stage enucleation of infused human erythroblasts, especially at 16 and 24 h post infusion. The quantitative analysis of four independent experiments is shown as the mean ± SD (*p* = 0.0307 and *p* = 0.0353 for 16 and 24 h, respectively). **I** Representative confocal microscopy images of enucleated cells sorted using the human CD235a surface marker from recipient mouse PB. No difference in morphology or CD235a expression was detected between the miR-125b-5p overexpression group and the control group.

More than 90% of cells from mouse embryonic Day 13.5 (E13.5) fetal livers were CD71^+^Ter119^+^, and most of these cells were nucleated erythroid cells as indicated by SYTO16/Ter119 staining (Fig. S9A and S9B). Similar to the findings in human erythroblasts, overexpression of miR-125b-5p increased the enucleation of these mFL-derived erythroblasts in vitro (Fig. S9C). To examine the in vivo function of miR-125b-5p, we transfected these cells with a miR-125b-5p mimic, labeled them with CFSE and transfused them into allogenic ICR mice (Fig. 4A). PB CFSE^+^ cells were detectable for 2 days in both the miR-125b-5p-overexpressing and control groups (Fig. 4B). At 24 h after transfusion, ~90% of CFSE^+^ cells were CD71^−^Ter119^+^ (Fig. 4C), indicating maturation of erythroblasts from both groups. The upregulation of miR-125b-5p facilitated enucleation, especially at 2 h and 16 h posttransfusion (Fig. 4D).

The in vivo maturation of hCB-MNC-derived erythroblasts in irradiated NOD/SCID mice showed similar results. On approximately induction Day 14, induced erythroblasts were predominantly CD235a^+^CD71^+^ and nucleated (Fig. 4E). After overexpression of miR-125b-5p (Fig. 4F) and cell transfusion, hCB-MNC-derived erythroblasts became fully differentiated and matured as indicated by an increased PB CD235a^+^CD71^−^ /CD235a^+^ ratio in both groups (Fig. 4G). Overexpression of miR-125b-5p accelerated enucleation at the early stage of infusion by 16 and 24 h (Fig. 4H). On posttransfusion Day 3, we sorted infused human cells by the expression of human CD235a. All isolated cells appeared similar to biconcave disks, which is the morphology of fully matured RBCs (Fig. 4I) [18].

### miR-125b-5p overexpression alters erythroblast microstructure

Throughout terminal erythropoiesis, erythroid progenitors undergo morphological changes, including decreases in cell size, nuclear condensation and cytoskeletal remodeling [3]. The alteration of actin arrangement is a key link in this process. In addition, loss of mitochondrial membrane potential following the clearance of mitochondria is also required for terminal erythroid maturation [27–29].

**Fig. 5:**
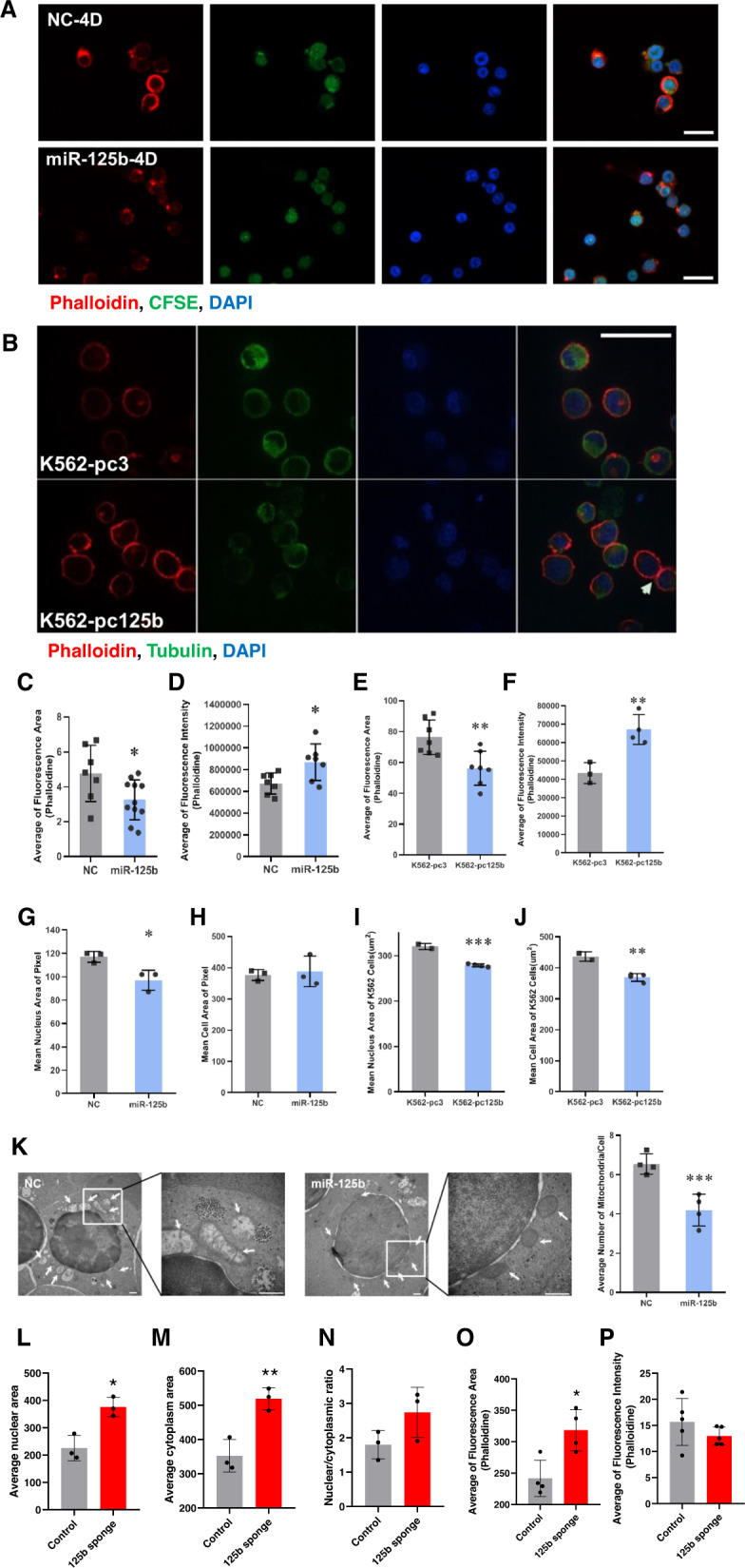
The upregulation of miR-125b-5p is highly correlated with actin rearrangement, nuclear condensation and mitochondrial clearance during the final stage of erythropoiesis. **A**–**J** Immunofluorescence staining and confocal microscopy indicating the status of actin (phalloidin, red), cytoplasm/CFSE and tubulin (green) and nuclei (DAPI, blue) in cells at the indicated induction times with or without miR-125b-5p treatment. Five random fluorescence images from each group were acquired and analyzed by HCS STUDIO software. Data from three independent experiments were statistically analyzed and are expressed as the mean ± SD. **A** hCB-MNC-derived erythroblasts on Day 4. Scale bars = 20 μm. **B** K562 cells after first-stage erythrocyte induction. Scale bars = 50 μm. **C**–**F** Overexpression of miR-125b-5p enhanced cytoskeletal remodeling. The average fluorescence area (**C**, *p* = 0.0329) and intensity (**D**, *p* = 0.0247) of phalloidin staining indicated actin rearrangement in hCB-MNC-derived erythroblasts. The average fluorescence area (**E**, *p* = 0.0056) and intensity (**F**, *p* = 0.0063) of phalloidin staining indicated actin rearrangement in K562 erythroid cells. **G**–**J** Cells with miR-125b-5p overexpression exhibited smaller nuclei. **G** Mean nuclear areas of hCB-MNC-derived erythroblasts (*p* = 0.0239). **H** Mean cell areas of hCB-MNC-derived erythroblasts. No significant difference was detected between the miR-125b-5p overexpression and control groups (*p* = 0.7225). **I** Mean nuclear areas of K562 erythroid cells (p = 0.0004). **J** Mean cell areas of K562 cells. Compared to control cells, miR-125b-5p-overexpressing cells exhibited a smaller cell size (*p* = 0.0045). **K**–**O** Seven days post AAV-miR-125b-sponge infection, immunofluorescence staining and statistical analysis indicated the alteration of nuclear shrinkage, cell size and the status of actin in mouse bone marrow erythroblasts. **K** Mean nuclear areas of BM erythroblasts, cells with miR-125b-5p KD exhibited larger nuclei. (*p* = 0.0112). **L** Mean cytoplasm areas of BM erythroblasts, cells with miR-125b-5p KD exhibited larger cytoplasm. (p = 0.0075) **M** Nuclear/ cytoplasmic ratio of BM erythroblasts, no statistically significant difference was detected (*p* = 0.1235). **N**, **O** miR-125b-5p KD affected cytoskeletal remodeling. The average fluorescence area (**N**, *p* = 0.0128) and intensity (**O**, *p* = 0.2476) of phalloidin staining indicated reduced actin aggregation after miR-125b-5p KD. **P** Representative images of electron micrographs of hCB-MNC-derived erythroblasts at enucleation Day 4 (left panel). The arrows indicate mitochondria. Scale bars=500 nm. miR-125b-5p overexpression destroyed mitochondrial structure and reduced the number of mitochondria. Statistical analysis of the mitochondrial number/cell from five random fields, and the results are expressed as the mean ± SD (right panel; *p* = 0.0021, **p < 0.01 and ***p < 0.001).

To examine actin filaments, we stained fixed hCB-MNC-derived erythroblasts (Fig. 5A) and K562 cells (Fig. 5B) at the indicated induction times with rhodamine-phalloidin, CFSE, anti-tubulin antibody and 4',6-diamidino-2-phenylindole (DAPI) for actin, cytoplasm, tubulin and nuclei imaging, respectively. After erythroid induction, the upregulation of miR-125b-5p facilitated actin aggregation in both primary erythrocytes and K562 cells, which was indicated by a contracted fluorescence area and enhanced fluorescence intensity (Fig. 5C–F). Overexpression of miR-125b-5p also promoted chromatin condensation (Fig. 5G and I) and showed a certain effect on cell mass reduction (Fig. 5H and J; only Fig. 5J shows a statistically significant difference).

Furthermore, electron microscopy imaging indicated that without alteration of mitochondrial perinuclear localization, miR-125b-5p overexpression resulted in a reduction in the number and volume of mitochondria as well as the destruction of the mitochondrial ultrastructure, suggesting the involvement of mitochondria in miR-125b-5p-mediated erythroid terminal maturation (Fig. 5K).

Erythroblasts from mouse bone marrow carrying AAV-miR-125b-sponge were also examined for the status of nuclear, cytoplasm and actin filaments. Bone marrow smear indicated that miR-125b KD increased erythroblast cell size (Fig. 5L) and nuclear size (Fig. 5M), the nuclear/cytoplasmic ration also showed a trend of enhancement although non-statistically significant (Fig. 5N and Fig. S10A). Enlarged phalloidin staining area (Fig. 5O) and reduced phalloidin fluorescence intensity (Fig. 5P and Fig. S10B) from bone marrow sections suggested reduced actin aggregation under miR-125b-5p KD.

### Bcl-2 is a direct target of miR-125b-5p during enucleation

**Fig. 6:**
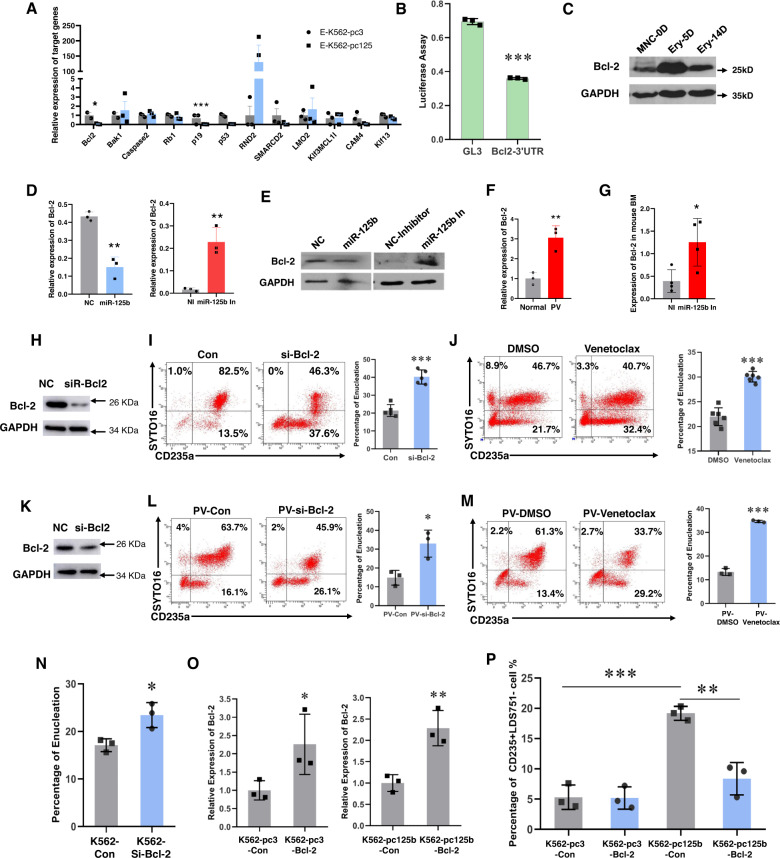
Downregulation of Bcl-2, a direct target of miR-125b-5p, is required for erythroblast enucleation. **A** The suppressive effect of miR-125b-5p on its putative targets in erythroid differentiated K562 cells was analyzed by qRT-PCR. The target candidates included apoptosis-related genes (Bcl-2, Bak1 and caspase-3), cell cycle-related genes (Rb1, p19 and p53), cytoskeleton-related genes (RND2 and SMARCD2), hematopoiesis-related genes (LMO2, KLF13 and MCL1) and an adhesion molecule (ICAM4). The relative expression was normalized to GAPDH (Data are expressed as the mean ± SEM. *p = 0.0277, ***p = 2.64E-06). **B** The relative luciferase activity indicated direct binding of miR-125b-5p to the Bcl-2 3'-UTR and its suppressive effect (*p* = 6.87068E-06). **C** Western blot showing the expression pattern of Bcl-2 at different stages of erythropoiesis. Quantitative RT-PCR (**D**) and western blot (**E**) analyses demonstrated that Bcl-2 expression was opposite to miR-125b-5p levels when miR-125b-5p expression was modulated with miRNA mimics or inhibitors (**p = 0.0014 and **p = 0.0048). **F** qRT-PCR analysis of the relative Bcl-2 expression in healthy controls and PV patient erythroblasts after enucleation induction. The quantification was normalized to GAPDH mRNA (*p* = 0.0061). **G** qRT-PCR showing the expression of Bcl-2 after miR-125b-5p inhibitor mimic intra-BM transfection into ICR mice. HPRT was used as the loading control (*p* = 0.0259). **H** The downregulation of Bcl-2 in hCB-MNC-derived erythroblasts using siRNA was detected with western blot. **I** Exogenous downregulation of Bcl-2 in hCB-MNC-derived erythroblasts enhanced the erythroblast enucleation rate. Data represent five independent experiments (*p* = 2.10622E-06). **J** Representative images of the flow cytometry assay and statistical analysis show that the Bcl-2 inhibitor venetoclax increased the erythroblast enucleation rate. Data represent six independent experiments (*p* = 2.75114E-06). **K** The downregulation of Bcl-2 in PV patient PB-MNC-derived erythroblasts using siRNA was detected with western blot. Representative images of flow cytometry assay and statistical analysis show that si-Bcl-2 (**L**) and venetoclax (**M**) increased the erythroblast enucleation rate of PV patients. Data represent three independent experiments (*p = 0.0186 and ***p = 1.82231E-05). **N** Statistical analysis of the K562 erythroid cell enucleation rate after Bcl-2 downregulation with siRNA (*p* = 0.0206; *n* = 3). **O** Rescued Bcl-2 expression by Bcl-2 vehicle transfection into miR-125b-5p-overexpressing K562 cells (K562-pc125) or expression control cells (K562-pc3). K562-pc3 cells with Bcl-2 expression vector or blank vector control were referred to as K562-pc3-Bcl-2 or K562-pc3-Con, respectively. K562-pc125 cells with Bcl-2 expression vector or blank vector control were referred to as K562-pc125b-Bcl-2 or K562-pc125b-Con, respectively. The expression of Bcl-2 was analyzed by qRT-PCR (*p = 0.0289 and **p = 0.0082). **P** Statistical analysis of the enucleation rate (CD235a^+^LDS751^-^%) of K562 erythroid cells under Bcl-2 rescue. Data represent three independent experiments and are expressed as the mean ± SD (***p = 0.0005 and **p = 0.003). In all qRT-PCR analyses, the results are expressed as the mean ± SD unless otherwise specified (*n* = 3; *p < 0.05, **p < 0.01 and ***p < 0.001).

Terminal stage miR-125b-5p modification showed no impact on cell cycle (Fig. S11). Excepted for GATA-1, key erythroid regulators were not consistently altered with miR-125b-5p upregulation or downregulation. (Fig. S12) One of the most significant effects of miR-125b-5p on terminal erythropoiesis is enucleation. To further understand how miR-125b-5p regulates erythroblast enucleation, we selected the potential targets of miR-125b-5p using the following criteria: predicted as miR-125b-5p targets by TargetScan; implicated in cytokinesis or mitochondrial signaling, which were indicated to be involved in enucleation based on erythroblast microstructure imaging; developmentally downregulated in erythroid terminal differentiation; and downregulated upon miR-125b-5p overexpression in the K562 cell erythroid differentiation system (Fig. 6A). Among the genes tested, Bcl-2 met all five criteria [6].

Bcl-2 is a well-known antiapoptosis gene that functions via the mitochondrial pathway. To determine whether Bcl-2 expression is regulated by miR-125b-5p, we performed a luciferase reporter assay. Compared to the transfection of empty vector (pc3), the transfection of primary miR-125b-5p coding sequences (miR-125b2) significantly lowered Bcl-2 3^'^UTR-merged luciferase activity (Fig. 6B). In contrast to miR-125b-5p, Bcl-2 was downregulated during erythroblast maturation (Fig. 6C). In addition, the trends in Bcl-2 expression at both the mRNA (Fig. 6D) and protein levels (Fig. 6E) was opposite that of miR-125b-5p under miR-125b-5p expression modulation with a miRNA mimic [6]. Interestingly, abnormal Bcl-2 expression was also observed in the pathological state. The relative expression of Bcl-2 was higher in MNC-derived erythroblasts from PV patients than in those from healthy controls (Fig. 6F). The direct downregulation of miR-125b-5p by intra-BM injection of its inhibitor in ICR mice resulted in higher Bcl-2 expression upon miR-125b-5p suppression (Fig. 6G). These findings imply a direct regulatory role of miR-125b-5p on Bcl-2.

The effect of Bcl-2 on erythroblast enucleation was further verified by direct Bcl-2 suppression with siRNA and small molecules. In hCB-MNC-derived erythroblasts, Bcl-2 knockdown with siRNA (Fig. 6H) doubled the erythroblast enucleation rate (Fig. 6I). Treatment with venetoclax, a Bcl-2-specific inhibitor and FDA-approved leukemia therapy drug, [30–32] also markedly promoted the enucleation of hCB-MNC-derived erythroblasts (Fig. 6J). Strikingly, Bcl-2 downregulation with shRNA (Figs. 6K, L) or venetoclax (Fig. 6M) also benefited the enucleation of PV patient-derived erythroblasts, which was consistent with the outcome of miR-125b-5p overexpression. For K562 cell erythropoiesis, the enucleation rate was also enhanced by Bcl-2 siRNA transfection (Fig. 6N). In contrast, the rescue of Bcl-2 expression in miR-125b-5p-overexpressing K562 cells with transfection of the Bcl-2 expression vector (Fig. 6O) decreased the enucleation efficiency (Fig. 6P). Therefore, miR-125b-5p may function in erythroblast enucleation by directly suppressing Bcl-2 expression.

### The miR-125b-5p-Bcl-2-ROCK axis plays an important role in erythroblast enucleation

**Fig. 7:**
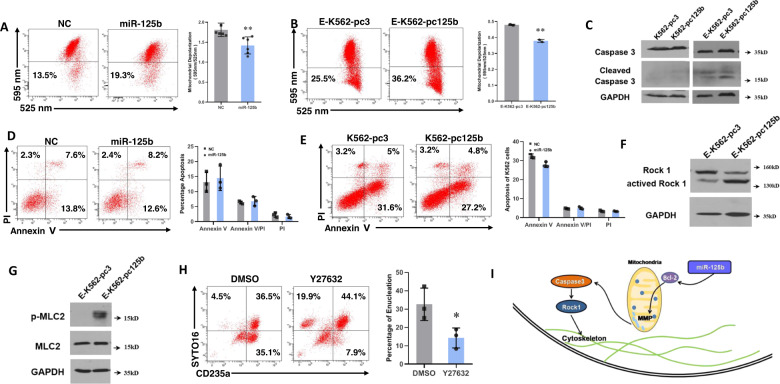
The miR-125b-5p-Bcl-2-ROCK signaling pathway is involved in erythroblast enucleation. Mitochondrial depolarization was determined by JC-1 staining and the ratio of the fluorescence intensity at 595 nm/525 nm in erythropoiesis of hCB-MNC-derived erythroblasts (**A**) and K562 cells (**B**) Representative images of flow cytometry analysis and statistical analysis of JC-1 staining are presented in the left and right panels, respectively. Statistical analysis of data from independent experiments is presented as the mean ± SD (A, *p* = 0.0054 (*n* = 6); and B, *p* = 0.0051 (*n* = 3)). **C** Representative images of western blots show caspase-3 and cleaved caspase-3 levels in miR-125b-5p-overexpressing/control K562 erythroid cells before and after erythroid differentiation. Cleaved caspase-3 was increased in erythroid differentiated K562 cells (E-K562). miR-125b-5p overexpression further enhanced cleaved caspase-3. **D**, **E** Representative images of flow cytometry assay (left panel) and statistical analysis (right panel) of apoptosis by Annexin-V/PI staining in hCB-MNC-derived erythroblasts (**D**) and erythroid K562 cells (**E**). Quantitative analysis of three independent experiments is shown as the mean ± SD. **F** Representative western blot images show ROCK-1 and active ROCK-1 levels in miR-125b-5p-overexpressing/control K562 cells after erythroid differentiation. **G** Representative western blot images show that the overexpression of miR-125b-5p in K562 erythroid cells improved phosphorylated MLC2 levels. **H** Flow cytometry analysis of primary erythroblast enucleation efficiency (CD235a^+^SYTO 16^−^%) after treatment with Y27632, a small molecule inhibitor of ROCK-1. Right panel, Statistical analysis of the enucleation rates of three independent experiments is expressed as the mean ± SD (*p* = 0.0138). **I** Working model of miR-125b-5p in erythroblast enucleation. miR-125b-5p downregulates the expression of Bcl-2 followed by mitochondrial depolarization and the activation of caspase-3. ROCK-1 is subsequently activated, which results in cytoskeletal rearrangement and efficient enucleation (*p < 0.05 and **p < 0.01).

To support a connection between miR-125b-5p-Bcl-2 and erythroblast enucleation, we investigated the downstream signaling of Bcl-2. Mitochondrial depolarization was first examined by JC-1 staining in K562 and hCB-MNC erythroid induction systems. Before induction, miR-125b-5p overexpression showed no effect on mitochondrial membrane potential in K562 cells (Fig. S13). Regarding the erythroid system, however, overexpression of miR-125b-5p enhanced the depolarization of mitochondria in both primary erythroblasts (Fig. 7A) and K562 cells (Fig. 7B) followed by the activation of caspase-8, caspase-9 (Fig. S14) and caspase-3 (Fig. 7C). To determine the involvement of caspase-3-induced apoptosis, we examined hCB-MNC-derived erythroblasts and K562 erythroid cells with Annexin-V/PI staining. miR-125b-5p overexpression resulted in no differences in apoptosis in either hCB-MNC-derived erythroblasts or K562 cells compared to controls (Fig. 7D and 7E) suggesting that apoptosis is not a designated consequence of miR-125b-5p signaling during erythroblast maturation.

Similar to a previous report, [33] cleaved caspase-3 activated the Rho-associated kinase ROCK-1 (Fig. 7F) in K562 erythroid cells, which contributed to the phosphorylation of myosin light chain 2 (MLC2) (Fig. 7G). Phosphorylation of MLC2 may induce its interaction with actin and activate myosin ATPase, resulting in enhanced cell contractility. The latter events are responsible for asymmetric cytokinesis and enucleation [34]. Moreover, the addition of Y27632, a small molecule inhibitor of ROCK-1, severely suppressed the enucleation of hCB-MNC-derived erythroblasts (Fig. 7H), thereby indicating ROCK-1 signaling during enucleation.

In conclusion, we demonstrated that caspase-3 activation followed by ROCK-1 activation is the downstream effect of Bcl-2 suppression, which is a consequence of miR-125b-5p overexpression. In addition, ROCK-1 activation followed by MLC2 phosphorylation may contribute to actin polymerization and terminal erythrocyte maturation/enucleation (Fig. 7I).

## Discussion

In the present study, the unique role of “ubiquitous” miR-125b-5p in erythroblast enucleation was discovered. Through endogenous miR-125b-5p expression analysis and gene gain- and loss-of-function studies, we confirmed the positive role of miR-125b-5p in erythroblast enucleation. We hypothesized that during physiological erythroblast maturation, miR-125b-5p accumulates to provide a final push for enucleation and that Bcl-2 is a mediator of gene function with the final outcome being cytoskeletal rearrangement and enucleation. Because in vitro-induced RBCs, which are separated from their common microenvironment, exhibit poor enucleation, [3] exogenous miR-125b-5p addition may promote enucleation and mature RBC production.

miR-125b-5p has shown functions in various stem/progenitor cells, cancer cells, and mature cells. Verified targets of miR-125b-5p include regulators involved in cell proliferation, apoptosis, differentiation, migration, epithelial-mesenchymal transition, metastasis and immune response [35–38]. Sun et al. suggested that the intracellular context determines miR-125b-5p behavior [39]. Our previous study also demonstrated that although miR-125b-5p plays a positive role during megakaryogenesis, it only exerts its defined functions when lineage determination has been fulfilled [40]. In the present study, by comparing PV and normal erythropoiesis, we demonstrated that although miR-125b-5p expression was high in PV cells before the basophilic erythroblast stage, it was not sufficiently upregulated afterward. The overexpression of miR-125b-5p did not alter erythroid lineage determination, which was consistent with a previous report, [10] but insufficient miR-125b-5p accumulation hampered erythroblast enucleation. Thus, late-stage miR-125b-5p accumulation may be important for erythroid terminal maturation. Direct erythroblast miR-125b-5p inhibition also verified the positive role of miR-125b-5p in enucleation. Accordingly, we deduced that miR-125b-5p is required for terminal erythroblast maturation, prompting us to modulate the miR-125b-5p gene to confirm its function during the stage when most erythroid cells are CD235a and CD71 double positive, i.e., basophilic erythroblasts [25].

At the terminal stage of erythropoiesis, erythrocytes undergo chromatin condensation before nuclear extrusion, and most of the gene transcription is gradually shut down by this stage [3]. To determine the key target of miR-125b-5p during enucleation, we examined the commonly accepted regulators of enucleation. Apoptosis, asymmetric cytokinesis, epigenomic regulation and vesicle trafficking are generally accepted enucleation theories [1, 2]. The overlap of predicted miR-125b-5p targets and putative enucleation regulators was examined, and Bcl-2, an apoptosis-related gene, was confirmed to play an important role in mediating miR-125b-5p functions. Increased miR-125b-5p expression in terminal erythroblasts resulted in Bcl-2 downregulation and was correlated with mitochondrial membrane potential reduction followed by caspase-3 activation. As previously reported, during late-stage erythropoiesis, the consequence of these apoptotic signals is not apoptosis but instead the activation of cytoskeletal regulators followed by the formation of the cytoplasm shrinkage ring [17, 33]. In the present study, the intracellular response included ROCK-1 (a cytoskeletal regulator) activation and MLC2 phosphorylation, both of which induce erythroblast enucleation. This response is similar to the consequence of another proapoptotic signal, Fas, which provides a positive stimulus for erythroid maturation without altering cell proliferation and apoptosis [33]. Due to the complexity of miR-125b-5p targets, there may be other regulators that mediate miR-125b-5p function during enucleation. Changes in mitochondrial morphology and function suggested that miR-125b-5p might function in enucleation through mitochondrial autophagy or metabolic regulation [41–43]. The upregulation of RND2, a member of the Rho family of GTPases, and the downregulation of p19^Ink4D^, a cell cycle regulator (Fig. 6A), also suggested the involvement of other regulatory methods or signaling pathways during miR-125b-5p function in enucleation.

To investigate the correlation between miR-125b-5p and erythroblast enucleation, we first examined the pathology of patients with PV. In addition to the emergence of NRBCs in PB, which may have resulted from extramedullary hematopoiesis or premature release, the enucleation rate of patients with PV was low even in BM CD235^+^CD71^−^ erythrocytes (Fig. 1D). Higher miR-125b-5p levels in PB leukocytes may be responsible for the morbidity of PV [14]. Similarly, miR-125b-5p was overexpressed in MNCs isolated from PV PB samples. However, distinct from the significant upregulation of miR-125b-5p near the normoblast stage in healthy erythropoiesis, the level of miR-125b-5p in patients with PV was insufficiently augmented during the late stage of erythropoiesis. Overexpression of miR-125b-5p in PV patient-derived EPCs enhanced the enucleation efficiency to normal levels. Thus, insufficient miR-125b-5p accumulation may be one of the leading causes of redundant NRBCs detected in the PB of patients with PV. Although the upstream signal of miR-125b-5p remains unknown, the synchronous dysfunction of JAK2 in both DS and PV may influence the role of miR-125b-5p in enucleation pathology [14, 44]. Further studies are required to determine the regulators of miR-125b-5p.

Taken together, these findings revealed the novel role of miR-125b-5p in the regulation of enucleation, the final step of erythropoiesis. The miR-125b-5p-Bcl-2-ROCK axis balances apoptosis and cytokinesis signaling, and it is implicated in erythroblast enucleation. Our study provides new insights into NRBC pathogenesis and in vitro RBC synthesis. Although targeted therapy with miR-125b-5p may not be applicable using current technology, gaining an understanding of the miR-125b-5p signaling pathway during erythrocyte maturation may help identify a solution for erythroid diseases. Regarding RBCs manufactured from stem cells, the small molecules involved in the enucleation signaling pathway as well as miR-125b-5p mimics may overcome the procedural bottleneck to allow the production of high-quality RBCs to meet the clinical demand for “man-made” blood.

## Materials and methods

### Cell culture

#### hCB-MNCs, HSCs, PB-MNCs and BM-MNCs

After informed consent was obtained, human CB, PB and BM were collected from healthy volunteers and PV patients. The Research Ethics Committee of Beijing Institute of Transfusion Medicine approved all the studies of MNCs, HSCs and differentiated cells. hCB-, PB- and BM-derived MNCs and HSCs were isolated as previously described [7]. Erythroid induction was performed using a step-wise protocol modified from Ulirsch et al. [45] Briefly, 5 × 10^6 ^mL^−1^ cells were cultured in differentiation medium containing StemSpan Serum-Free Expansion Medium (SFEM, STEMCELL Technologies, Vancouver, Canada) supplemented with 100 ng mL^−1^ stem cell factor (SCF, PeproTech, Rocky Hill, NJ, USA), 40 ng mL^−1^ insulin-like growth factor 1 (IGF-1, R&D Systems, Minneapolis, MN, USA), 100 μg mL^−1^ holo-human transferrin (Sigma-Aldrich, St. Louis, MO, USA), 5 IU mL^−1^ erythropoietin (Epo, Kyowa Hakko Kirin, Tokyo, Japan), 1 μM dexamethasone (Sigma-Aldrich), 40 ng mL^−1^ lipid (Sigma-Aldrich) and 2 mM glutamine (Gibco, Big island, NY, USA) for 14–16 days. Next, in the terminal enucleation step, 5 × 10^5 ^mL^−1^ cells were cultured in the presence of 300 μg mL^−1^ holo-human transferrin (Sigma-Aldrich), 10 μg mL^−1^ recombinant human insulin (Sigma), 3 IU mL^−1^ heparin (STEMCELL Technologies) and 5% AB plasma (Atlanta Biologicals, Lawrenceville, GA, USA) in Iscove’s Modified Dulbecco’s Medium (IMDM, Gibco).

#### Human erythroleukemia K562 cells

Human erythroleukemia K562 cells were grown in basic medium containing RPMI-1640 supplemented with 10% fetal bovine serum. The 2-step differentiation procedure was: first, 1 × 10^5 ^mL^−1^ cells were cultured in basic medium supplemented with 40 μM Hemin (Sigma-Aldrich) and 100 ng mL^−1^ ara-C (Sigma-Aldrich) for 6 days; second, 1 × 10^5 ^mL^−1^ cells were suspended in basic medium with 40 μM Hemin (Sigma-Aldrich) and 14 µl mL^−1^ dimethyl sulfoxide (DMSO, Sigma-Aldrich) for 4 days.

#### Mouse fetal liver (mFL)-derived erythroblasts

Erythroblasts were purified from E13.5 mFL cells and cultured and differentiated as previously described [6].

### Cytospin preparation

May-Grunwald (MG500, Sigma-Aldrich) and Giemsa solution (GS500, Sigma-Aldrich) were used as previously described [46].

### Mouse model studies

Animal experiments were approved by the Beijing Medical Experimental Animal Care Commission (IACUC of AMMS-2014-036), and the procedures were in strict accordance with the Academy of Military Medical Sciences for the Care and Use of Laboratory Animals.

#### ICR mouse model for miR-125b-5p inhibitor injection

Administration of 50 μg miRNA inhibitor mimics or negative control mimics (Table S2) per mouse was achieved by intra-BM injection together with Entranster^TM^-in vivo (Engreen, Beijing, China) and glucose in vivo. After 40 h, the operation was repeated once. Twenty-four hours after the second injection, cells from PB, BM and spleen were harvested, washed and co-labeled with antibodies.

#### Transduction of BALB/c mice with AAV carrying a miR-125b-5p sponge

To specifically knockdown miR-125b-5p in erythroblasts, BALB/c mice were transduced with adeno-associated virus (AAV) serotype 6 which carried beta globin enhancer (GenBank: S73747.1) and promoter (GenBank: GU057255.1) [47] driving enhanced green fluorescent protein (eGFP) together with either miR-125b sponge (8-repeat antisense-miR-125b-5p) or control (8-repeat scramble nucleotides). The designed enhancer-promoter-eGFP with 8 tandem repeat miR-125b-5p sponges (5′-TCACAAGTTAGGGTCTCAGGGA-3′) or a control (5′-AAGTTTTCAGAAAGCTAACA-3′) [48] was cloned into AAV by Genechem Co. Ltd. (Shanghai, China). AAVs (1 × 10^11^ v.g) were tail vein injected into each mouse, and the effect of viral transfusion was followed for 7 days.

#### ICR mouse model for mFL-derived erythroblast transplantation

ICR mice (5–6 weeks of age) were initially irradiated with 6 Gy by a cobalt-60 source (1.096 Gy/min). Six hours later, mFL-derived erythroblasts (7.5 × 10^6^ cells/mouse), which were washed and labeled with CFSE (Invitrogen, Carlsbad, CA, USA), were injected through the caudal vein. The erythroblasts were transfected with miR-125b-5p or control mimics (Table S2) and cultured for 3 days before injection. At defined time points, PB cells were harvested, washed and co-labeled with LDS-751, anti-CD71 and anti-Ter119 antibodies.

#### NOD/SCID mouse for human erythroblast transplantation

For MNC-derived erythroblast transplantation, cells were prepared and transfected with miR-125b-5p or NC mimics and cultured for 3 days. The recipient mice (5–6 weeks of age) were initially conditioned by sublethal irradiation with 3.5 Gy from a cobalt-60 source (1.091 Gy/min), followed by intravenous injection of 1 × 10^7^ cells/mouse. At designated time points, PB cells were harvested, washed and co-labeled with LDS-751, anti-CD71 and anti-CD235a antibodies. On day 3, CD235a^+^ cells were sorted and examined by confocal laser scanning microscopy.

### Plasmid, miRNA mimic and inhibitor transfection

#### Plasmid transfections

Pri-miR-125b2 coding sequence was transfected into K562 cells through a pcDNA3.1-neomycin vector. Stable transfections were selected with 500 μg mL^−1^ G418. Bcl-2 coding sequence (pCEP4 Bcl-2, #16461, Addgene, Cambridge, MA, USA) was transfected and selected with 50 μg mL^−1^ hygromycin B.

### miRNA mimic and inhibitor transfection

According to the manufacturer’s instructions, cells were seeded in 6-well plates and transfected with 3 μg per well miRNA mimics (Genepharma, Shanghai, China) or non-target NC mimics (Table S2) with the aid of Entranster^TM^-R4000 (Engreen).

### Luciferase reporter assay

K562-pc125b cells or K562-pc3 cells (6 × 10^4^) were co-transfected with 8 ng pRL Renilla luciferase vector (Promega, Madison, WI, USA) as internal control and 800 ng pGL3-Bcl-2-3'UTR or pGL3 vector control. The cells were harvested 48 h post transfection and evaluated their luciferase activity using the dual-luciferase assay kit according to the manufacturer’s instruction (Promega). The luciferase activity in miR-125b-5p stably overexpressed cells (K562-pc125b) were normalized to vector transfection control (K562-pc3 cells).

### Quantitative RT-PCR (qRT-PCR)

qRT-PCR was carried out as previously reported [49]. Gene expression was normalized to GAPDH or HPRT. miRNA expression relative to U6 snRNA and cell numbers was assayed using All-in-One qPCR Mix (GeneCopoeia, Rockville, MD, USA). Error bars represent the standard deviation (SD), and the results are expressed as the mean ± SD. The primers are listed in supplementary material Table S1.

### Western blots

Western blots were performed by using antibodies against human Bcl-2 (Santa Cruz, Santa Cruz, CA, USA), GAPDH (Earth Ox, Millbrae, CA, USA), Caspase-3 (Cell Signaling Technology, CST, Danvers, MA, USA), ROCK-1 (CST), MLC2 (CST) and p-MLC2 (CST). The blots were visualized using an ECL kit (Santa Cruz).

### Immunofluorescence

Cells were fixed in 4% paraformaldehyde for 15 min and treated with 0.125% Triton X-100 for 10 min. Fixed cells were then blocked with 10% serum for 30 min. Next, the cells were incubated with tubulin antibodies overnight at 4 °C. After three washes with phosphate-buffered saline (PBS), the cells were incubated with phalloidin together with the corresponding secondary antibody. Then, the cells were stained with DAPI for 2 min. Finally, stained cells were visualized by confocal microscope (PE Ultra VIEW VoX).

### Transmission electron microscopy (TEM)

The cells were firstly fixed with 4% paraformaldehyde and 1% glutaraldehyde for 48 h, then incubated with 1% osmium tetroxide for 1 h, and dehydrated using series of ethanol solutions. The dehydrated cells were embedded in Polybed 812 epoxy resin (Polysciences, Warrington, PA, USA), ultrathin sectioned and collected on 200 mesh copper grids. The cell sections were stained with 4% aqueous uranyl acetate for 15 min, and with Reynolds’ lead citrate for 7 min. Then stained sections were examined using a H7650 transmission electron microscope (HITACHI, Tokyo, Japan).

### Fluorescence-activated cell sorting (FACS)

Cells were harvested and washed three times with PBS. Then, 5 μL per 1 × 10^6^ cells were stained with either anti-human CD71-APC (BD Biosciences, Franklin, NJ, USA) or anti-human CD71-FITC (BD Biosciences), anti-human CD235a-PE (BD Biosciences), anti-human α4 integrin (CD49d-PE, eBioscience, San Diego, CA, USA), anti-human Band 3-APC (graciously provided by Professor Xiuli An) or anti-mouse CD71-PE (eBioscience) and anti-mouse Ter119-APC at 4 °C for 40 min. Subsequently, the cells were washed with PBS or normal saline (NS), followed by analysis using a FACS Calibur machine (BD Biosciences).

#### Nuclear staining

Cell nuclei were detected by staining with LDS-751 (Invitrogen), SYTO16 Green Fluorescent Nucleic Acid Stain (Life technologies, Inc., Grand Island, NY, USA) or SYTO62 Red Fluorescent Nucleic Acid Stain (Life technologies). LDS751 was supplemented together with antibodies used in FACS at 2 μg mL^−1^ for K562 cells, BM and PB derived cells. Samples were washed with PBS and analysis with FACS Calibur machine (BD Biosciences). When SYTO16 or SYTO62 was used for human and mouse erythroblast enucleation detection, cell suspensions in NS were adjusted to 250 μL and analyzed with a FACS Calibur machine by adding 25 nM SYTO16/SYTO62 for at least 10 min.

### Assessment of mitochondrial membrane potential and cell apoptosis

Mitochondrial membrane potential was estimated with fluorescent dye JC-1 (Molecular Probes, Grand Island, NY, USA). According to the manufacturer’s instructions, briefly, 5 × 10^5^ cells were re-suspended in 1 mL fresh complete medium and incubated with JC-1 (2.5 mM) for 30 min at 37 °C in the dark. Then the cells were washed with PBS and analyzed using a flow cytometer with 488 nm excitation laser (BD Biosciences). The mitochondrial membrane potential was judged by the ratio of red (595 nm) to green (525 nm) fluorescence intensity.

For apoptotic testing, cells were quantified by Annexin-V/PI staining kit (Dojindo Laboratories, Kumamoto, Japan) and flow cytometry (BD Biosciences) analysis.

### Statistical analysis

Data are presented from at least three separate experiments and show as the mean ± SD. Two-tailed student’s *t*-test was used for significant differences evaluation.

## Supplementary information

supplementary imformation

Original Data File

Reproducibility checklist

## Data Availability

All data needed to evaluate the conclusions in the paper are present in the paper and/or the Supplementary Materials. Additional data related to this paper may be requested from the authors.
