# Supplementary material for: The accumulation of miR-125b-5p is indispensable for efficient erythroblast enucleation
Source: Cell Death Dis. 2022 Oct 21;13(10):886. doi: 10.1038/s41419-022-05331-5 (PMC9586935; doi:10.1038/s41419-022-05331-5)
Supplement: Supplementary file 1 — supplementary imformation [file 41419_2022_5331_MOESM1_ESM.docx]

**Supplementary Figures**

**
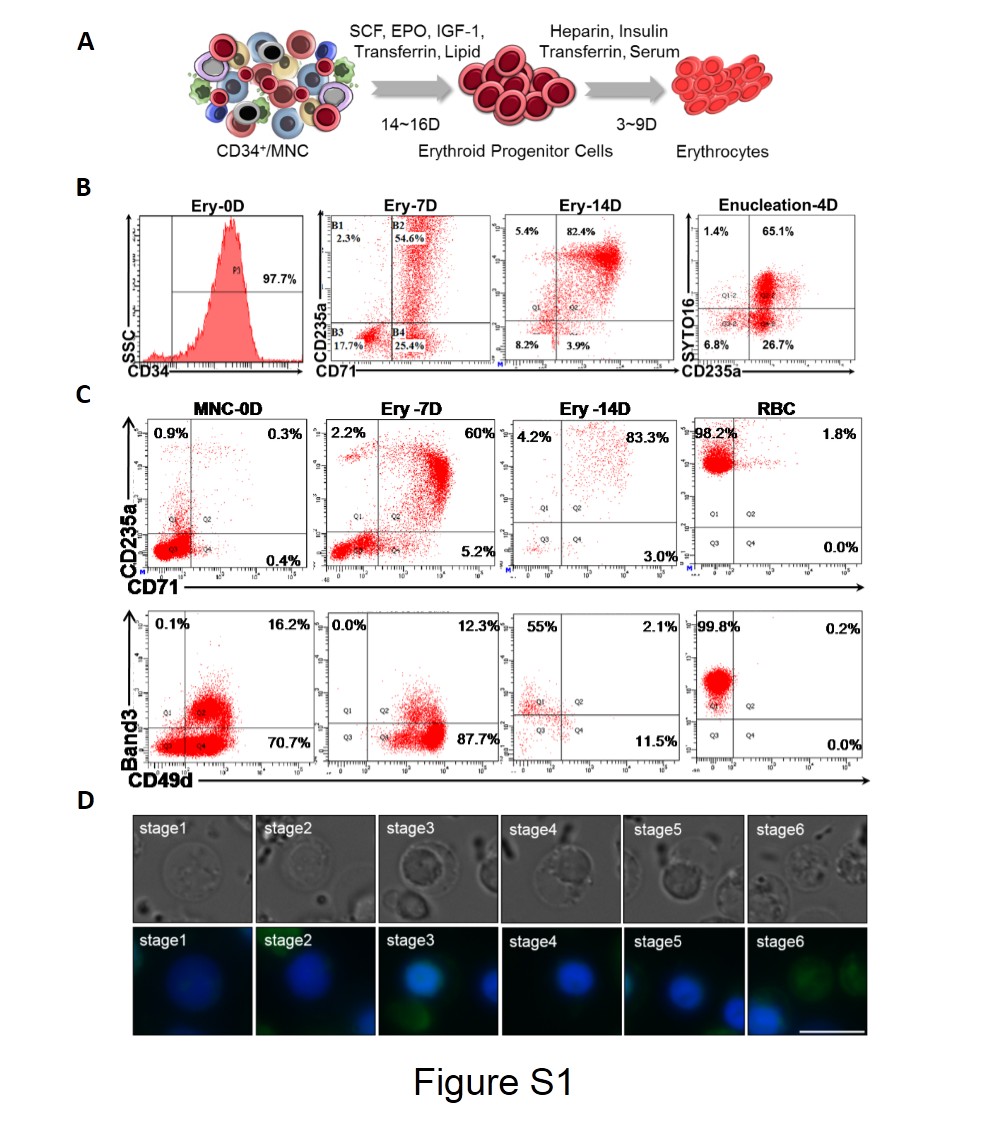
**

**Figure S1. A two-step hCB-CD34/MNC-Erythrocyte induction system.**

**(A)** Schematic illustration represents the two-step erythrocyte induction system. The first stage is for erythroid specific differentiation, and the second stage is for erythrocyte enucleation. **(B)** Flow cytometry analysis represents the expression of CD34, CD71 and CD235a in erythropoiesis on differentiation stage day 0 (Ery-0D), 7 (Ery-7D), 14 (Ery-14D) and on the enucleation stage day 4. **(C)** Representative images of flow cytometry analysis of CD71/CD235a and CD49d (α4-integrin)/Band3 expression during erythropoiesis with the induction system. **(D)** Confocal laser scanning shows the morphology of cells at different enucleation stage with the induction system. Scale bars: 10 μm.
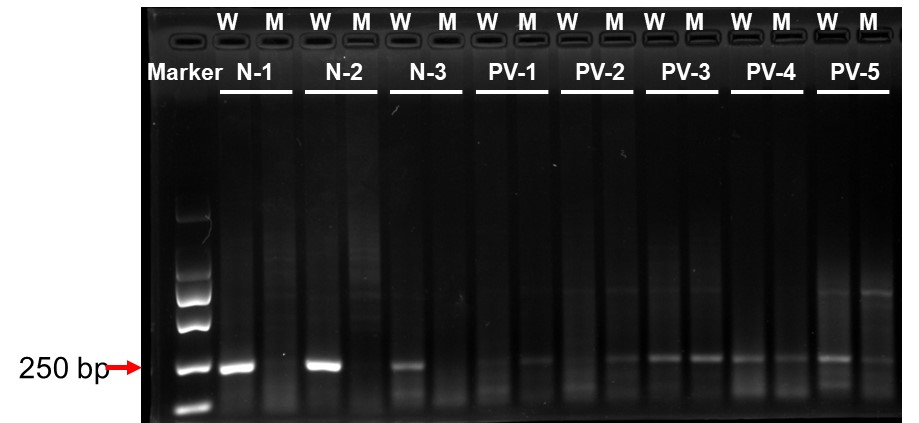


**Figure S2. JAK2 V617F mutation analysis.**

DNA samples were harvested from polycythemia vera (PV) patients’ or healthy controls’ peripheral blood mononuclear cells. PCR and electrophoresis were performed as previously described. ^1^ Bands amplified with mutant primers suggested the existent of JAK2 V617F mutation. W stands for wide type primer amplification, and M stand for mutant primer amplification. N stands for normal individuals and PV stands for individuals with polycythemia vera.

**
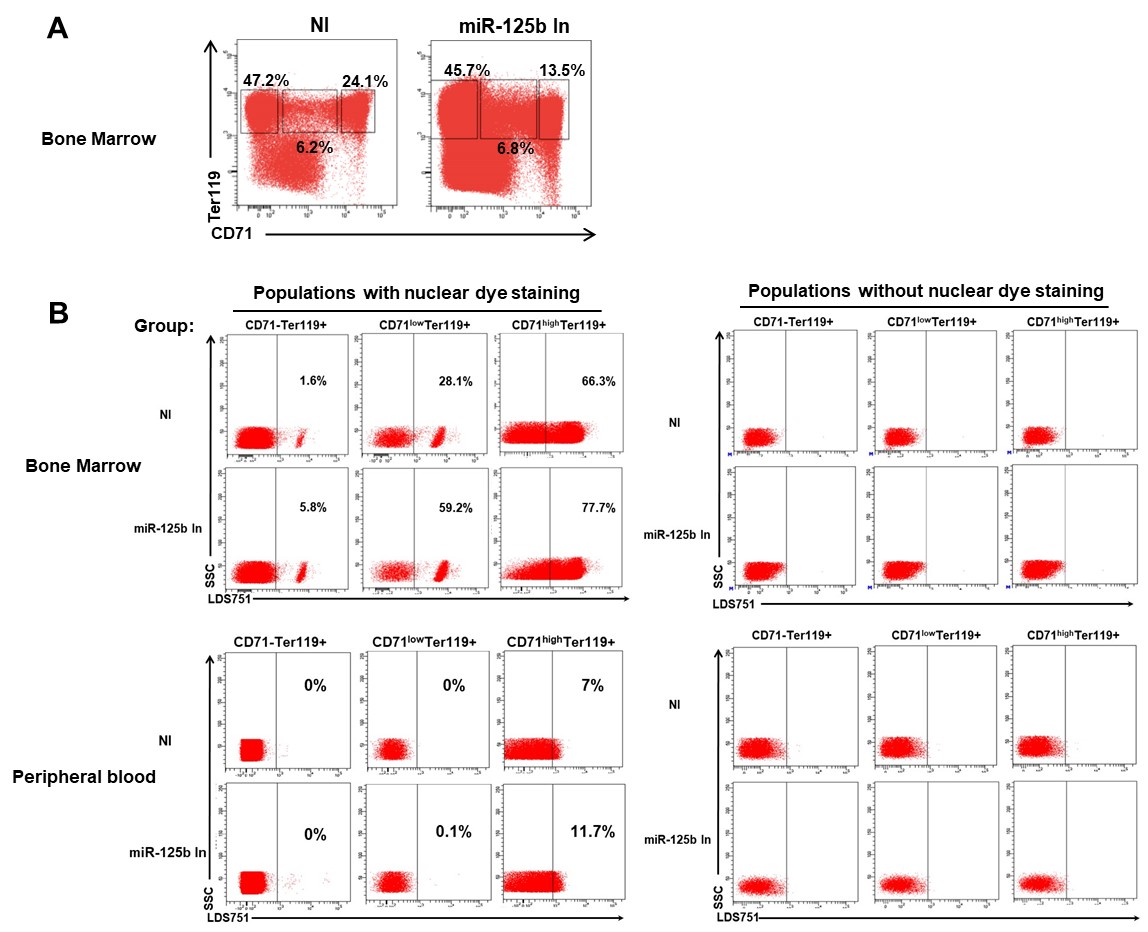
**

**Figure S3. Representative images of flow cytometry show nucleated populations from bone marrow and peripheral blood on miR-125b inhibitor injection day 3.** **(A)** CD71 and Ter119 antibody staining demonstrated the differentiation of bone marrow erythroblasts. **(B)** Enucleation analysis. Nuclear dye LDS751 unstaining controls are shown in the right panel.

**
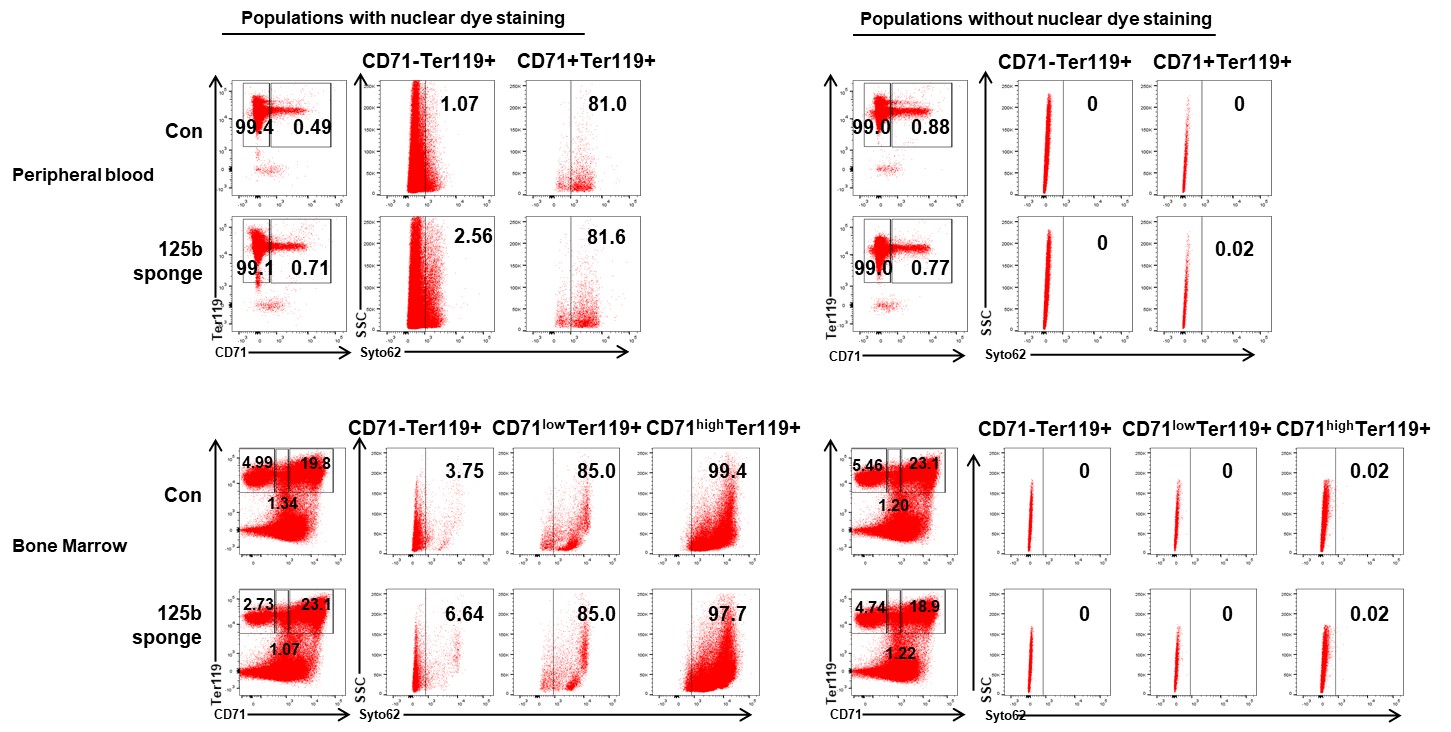
**

**Figure S4. Representative images of flow cytometry show nucleated populations from bone marrow and peripheral blood on miR-125b sponge injection day 7.** Nuclear dye SYTO62 unstaining controls are shown in the right panel.

**Figure S5. Colony-forming unit (CFU) assay shows the impact of miR-125b sponge injection on mouse bone marrow hematopoietic stem /progenitor cells.** Seven days after AAV-miR-125b sponge infusion, mouse bone marrow mononuclear cells (mMNCs) were harvested and plated in methylcellulose-based medium with recombinant cytokines (M3434, StemCell Technologies) for mouse hematopoietic cell CFU assays. Erythroblast specific miR-125b knockdown showed no effect on BM mMNC colony forming capability.

**
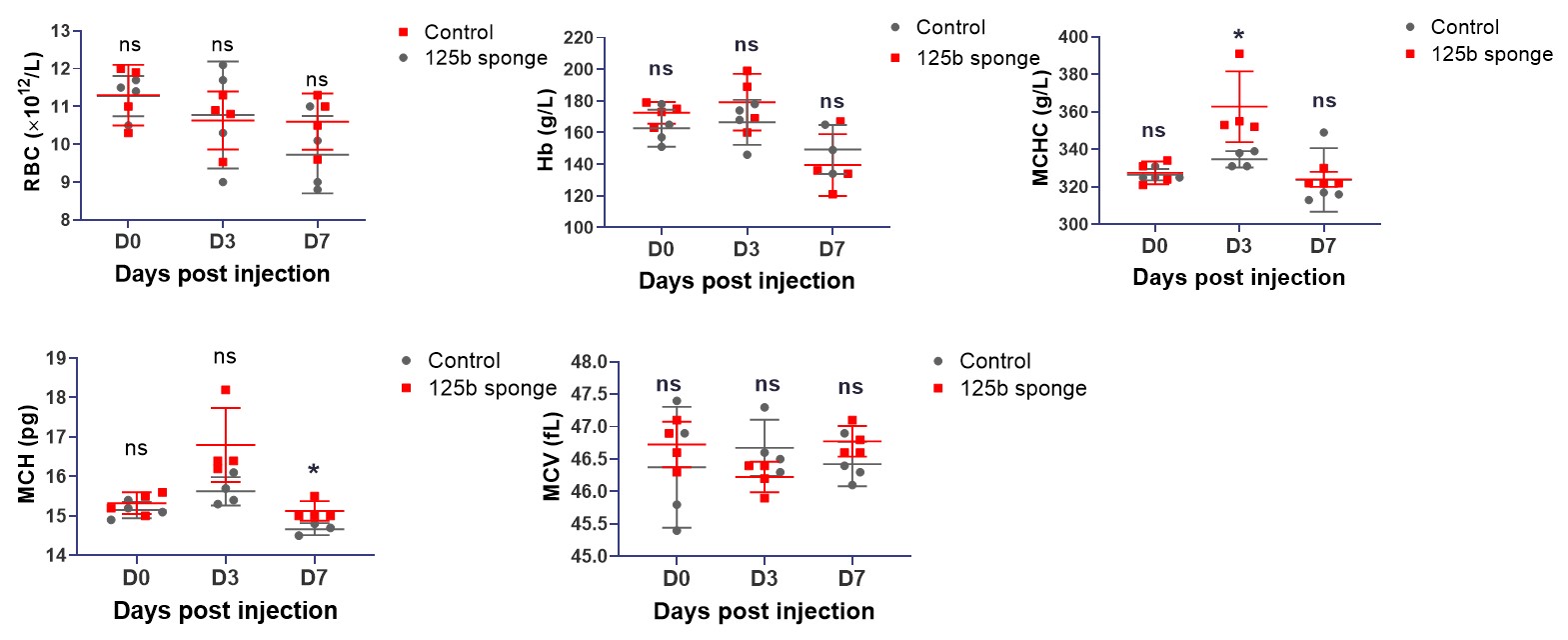
**

**Figure S6. Hemogram analysis of mice injected with AAV-miR-125b sponge.** 10 μL peripheral blood was harvested from mouse tail vein and loaded on an automated hematology analyzer for hemogram analysis.

**
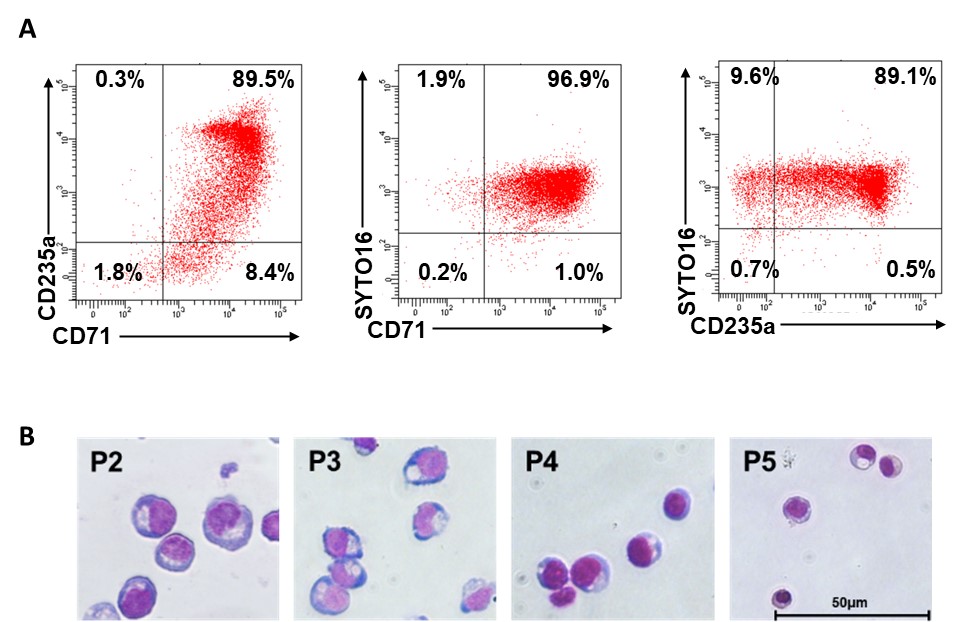
**

**Figure S7. Representative cytospin images of sorted erythroid populations based on the expression of CD71 and CD235a surface markers.** CD34^+^ hematopoietic stem/ progenitor cells were induced toward erythrocytes. On induction day 14, cells were analyzed by surface marker CD71 and CD235a and nuclear dye Syto16 **(A)**. Populations were gated as P2- CD71^+^CD235a^-^, P3- CD71^+^CD235a^med^, P4- CD71^+^CD235a^+^, and P5- CD71^-^CD235a^+^ **(B)**.

**
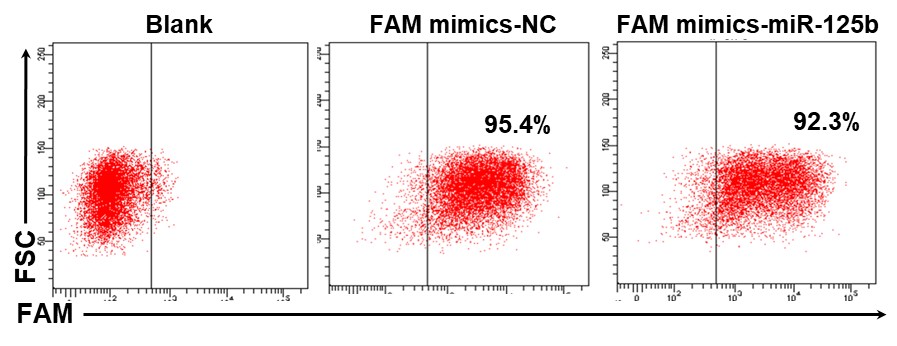
**

**Figure S8. Representative images of flow cytometry show miRNA mimics transfection efficiency in erythroblasts.**

**
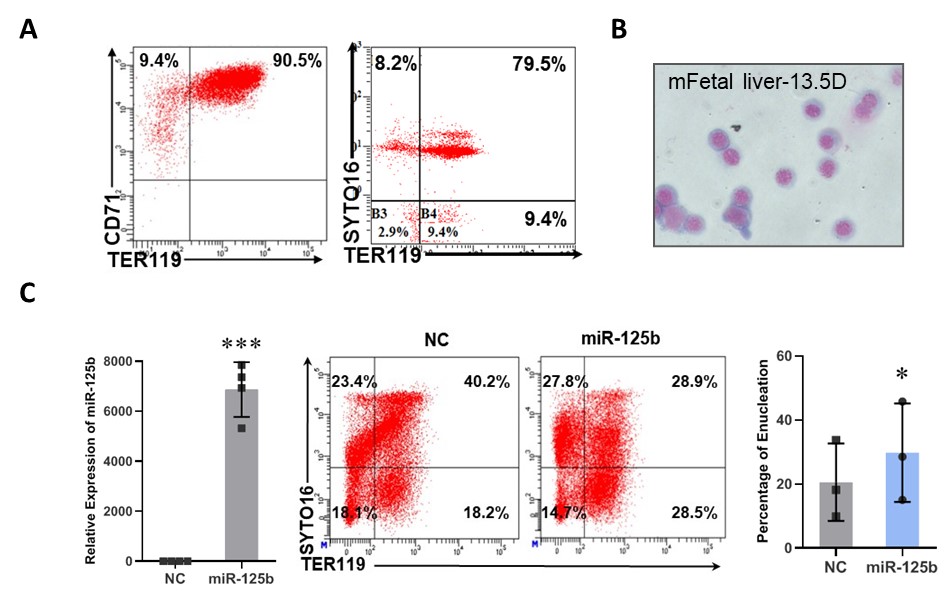
**

**Figure S9. miR-125b prompts cultured mouse fetal erythroblast enucleation.**

**(A)** Representative flow cytometry plots show the expression of CD71/TER-119 on E13.5 mFL derived cells. About 90% cells are nucleated. **(B)** Representative images display the morphology of mFL derived erythroblasts with May-Grünwald Giemsa staining. **(C)** The overexpression of miR-125b irritates enucleation efficiency in mFL derived erythroblasts. Left: qRT-PCR shows the relative expression of miR-125b after microRNA mimics transfection. p=1.58526E-05. Middle: Flow cytometry analysis of the TER-119^+^LDS751^-^ enucleated populations. Right: Statistical analysis of enucleation rate from three independent experiments is expressed as mean ± SD. p= 0.047.

**
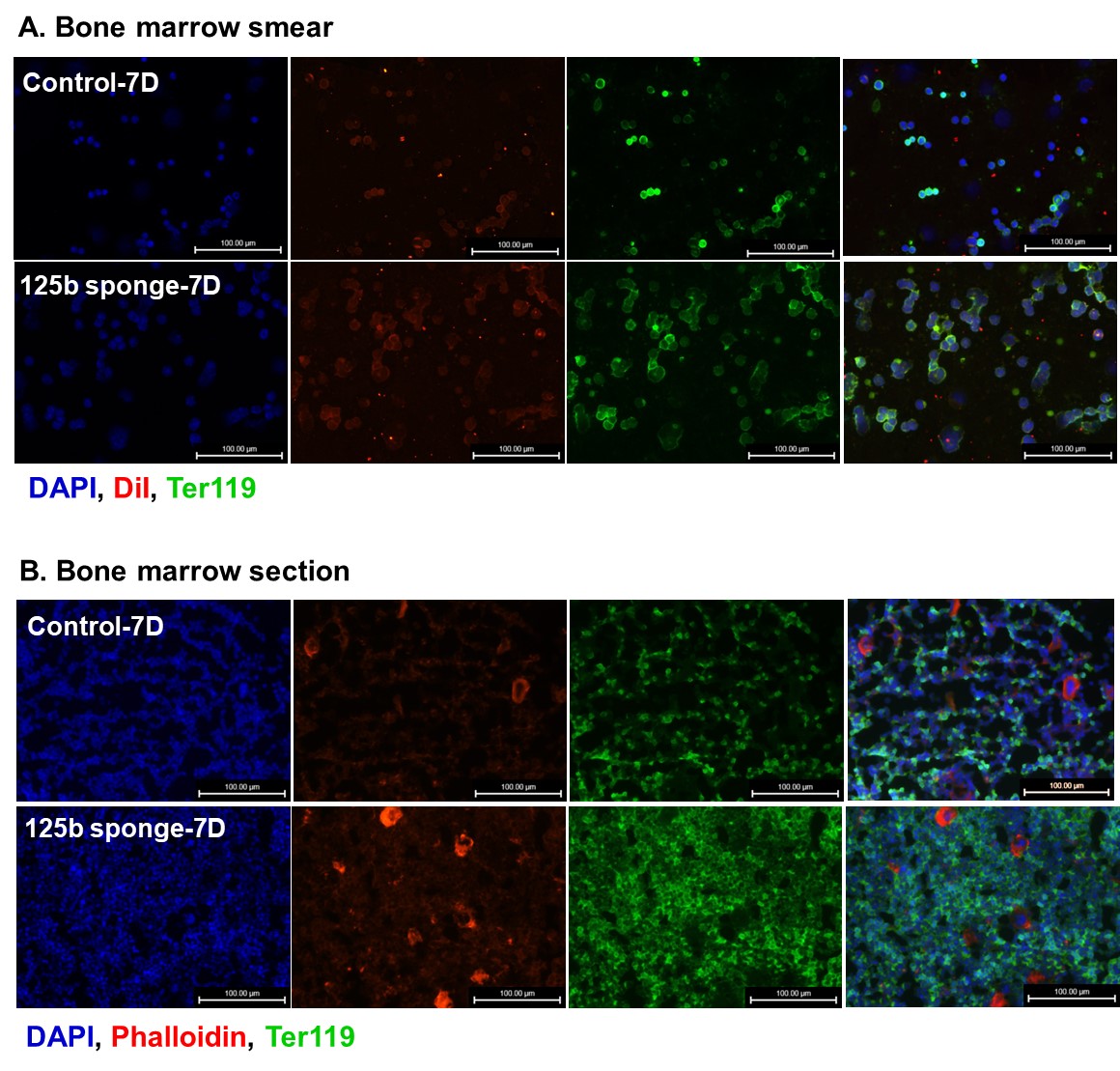
**

**Figure S10. Representative immunofluorescence images demonstrating the status of bone marrow erythroblasts 7 days post AAV-miR-125b sponge injection.** Mice injected with AAV-miR-125b sponge or control virus were sacrificed 7 days post injection. The bone marrow was washed out for smears and stained with Ter119 antibodies to indicate erythroblasts (green), and DAPI for nuclear (blue), DiI for cell membrane indication (red) respectively **(A)**. Bone marrow sections were stained with DAPI (nuclear, blue), phalloidin (actin, red) and Ter119 antibody (erythroblasts, green) respectively to demonstrate the status of actin during erythropoiesis **(B)**. Aipathwell software was applied for image analysis.

**
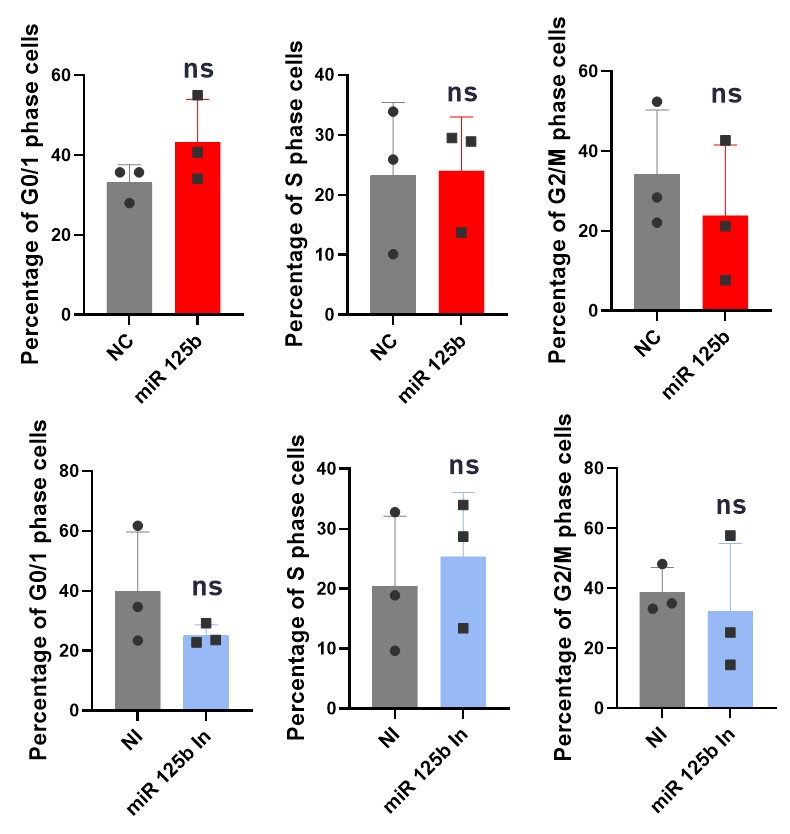
**

**Figure S11. Erythroblast cell cycle analysis afer miR-125b-5p mimics/inhibitor modification.** Cord blood mononuclear cells were induced toward erythroid lineage for 14 days, then transfected with miR-125b-5p mimics or inhibitors for miR-125b-5p overexpression or down-regulation. Five days after transfection, cell cycle status was interpreted using PI staining and flow cytometry analysis.

**
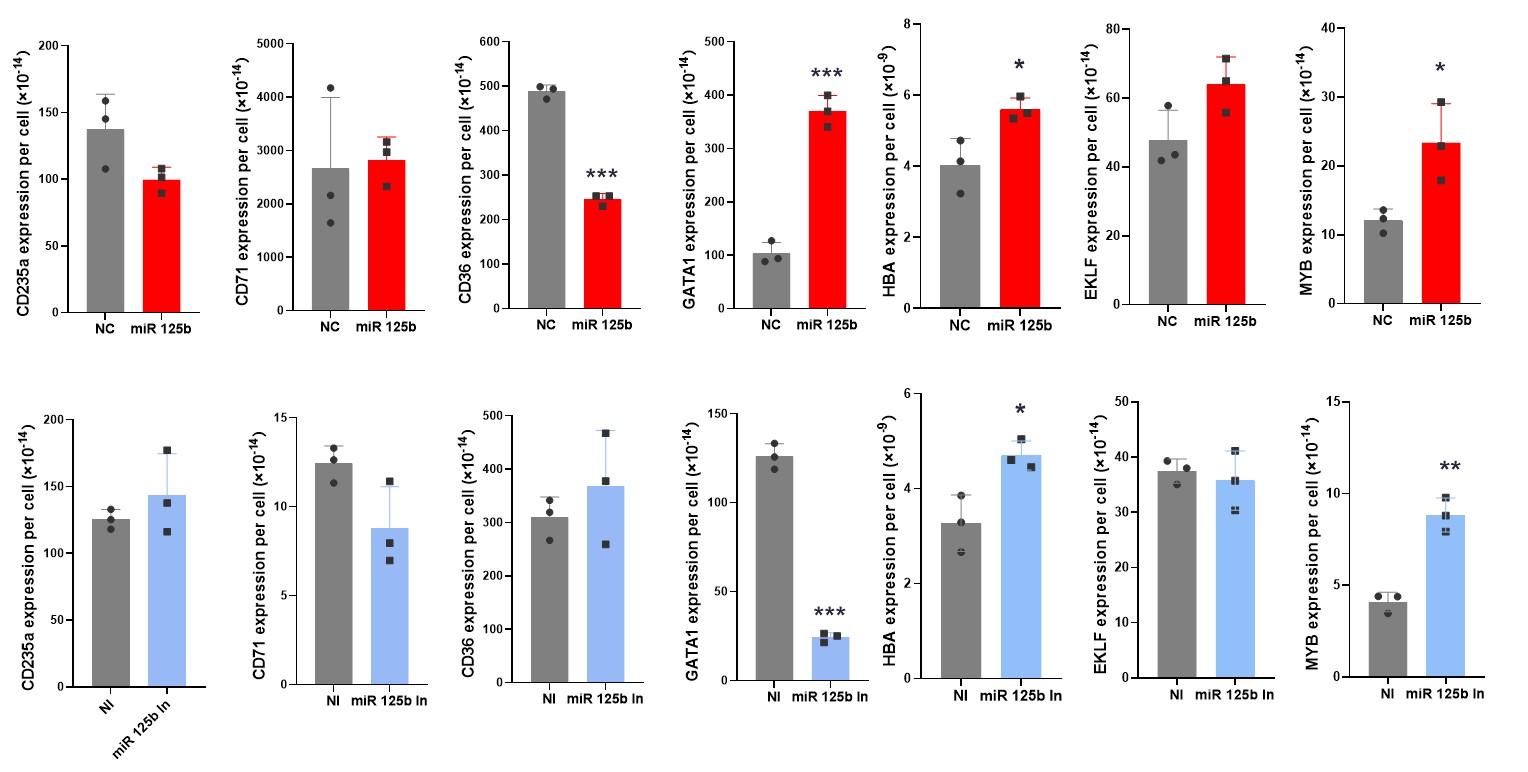
**

**Figure S12. Quantitative RT-PCR analysis for the expression of key erythroid genes after miR-125b-5p modification.** Cord blood mononuclear cells were induced toward erythroid lineage for 14 days, then transfected with miR-125b-5p mimics or inhibitors for miR-125b-5p overexpression or down-regulation. Five days after transfection, cells were harvested for quantitative RT-PCR analysis. All the gene expression was normalized with cell number.

**
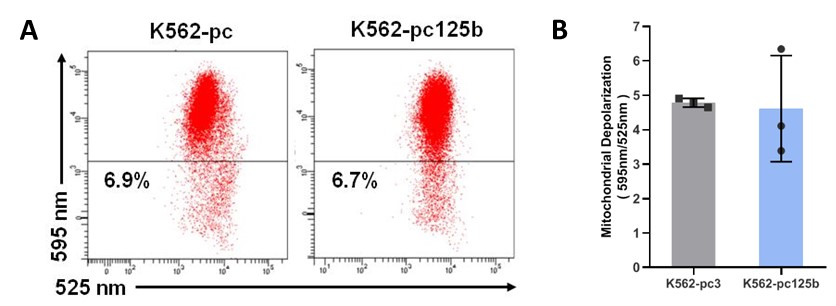
**

**Figure S13. In K562 cells, miR-125b overexpression shows no effect on mitochondrial membrane potential before erythroid induction.**

**(A)** Representative flow cytometry plots of JC-1 staining in K562 cells without erythroid induction. **(B)** Statistical analysis of data from three independent experiments is presented as the mean ± SD, p= 0.8585.


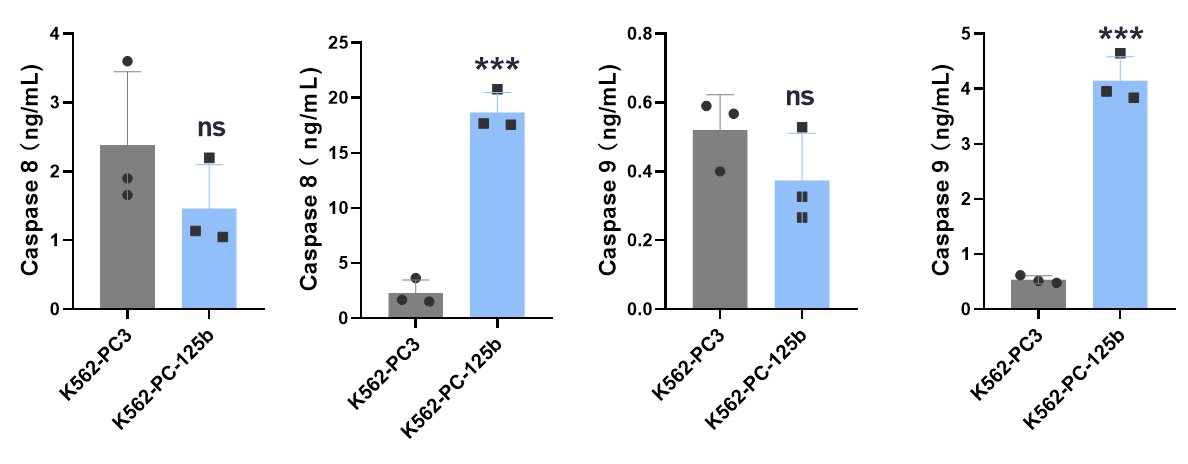


**Figure S14. Erythroid miR-125b-5p overexpression enhanced Caspase 8 and Caspase 9 activation.** Stable miR-125b-5p-overexpressing K562 cell line (K562-pc125b) and control cell line (K562-pc3) were subjected for Caspase activity analysis using human Caspase 8 or Caspase 9 ELISA Kit (Abclonal, Cat# RK01039, Cat# RK04177). The activation of Caspase 8 and Caspase 9 by miR-125b-5p overexpression was only detected after erythroid differentiation of K562 cells. **(A)** Caspase 8 activity detection before erythroid induction. **(B)** Caspase 8 activity detection after erythroid induction. **(C)** Caspase 9 activity detection before erythroid induction. **(D)** Caspase 9 activity detection after erythroid induction.

**Table S1. Primers used for qRT-PCR.**

| **Gene** | **Primers (forward, reverse)** |
| --- | --- |
| **miR-125b** | TCCCTGAGACCCTAACTTGTGA |
| **U6** | CGCTTCGGCAGCACATATACTA |
| **miUniv3** | GATTGAATCGAGCACCAGTTAC |
| **miR-125b** | ACTAGTGGATGGGTCATGGTGAAAAC |
|  | CTGCAGGTCTGGTTTGATGCGGTAGA |
| **JAK2V617F common R** | ATTGCTTTCCTTTTTCACAAGAT |
| **JAK2V617F wild type F** | GCATTTGGTTTTAAATTATGGAGTATATG |
| **JAK2V617F mutant F** | GCATTTGGTTTTAAATTATGGAGTATATT |
| **Bcl-2** | GAGGATTGTGGCCTTCTTTG |
|  | ATCACCAAGTGCACCTACCC |
| **Bak1** | CATCAACCGACGCTATGACTC |
|  | GTCAGGCCATGCTGGTAGAC |
| **Caspase2** | CTGACAGGGGACGCAGGATA |
|  | GCTCCCTCATTTCCAAGGTGA |
| **Rb1** | TTGGATCACAGCGATACAAACTT |
|  | AGCGCACGCCAATAAAGACAT |
| **p19** | CTGCAGGTCATGATGTTTGG |
|  | CAGCAGTGTGACCCTCTTGA |
| **p53** | GCAGCGCCTCACAACCTCCGTCAT |
|  | CCCCTCCTGGCCCCTGTCATCTTC |
| **RND2** | ACTGGACATGCGGACTGAC |
|  | CTGAGCGGATCGCTGCATT |
| **SMARCD2** | AAGCCTCTGACACAAAAGCGA |
|  | CTTCCACTCGGAGTTCCCA |
| **LMO2** | CCTTCAGAGGAACCAGTGGATG |
|  | TTCCGGCCCAGTTTGTAGTAGA |
| **Klf13** | GGAAATCTTCGCACCTCAAG |
|  | GGCAGCTGAACTTCTTCTCG |
| **MCL1** | GCCTCAAAAGAAACGCGGTAA |
|  | TCCGTAGCCAAAAGTCGCC |
| **ICAM4** | CCAACGTGACCTTGACCTACG |
|  | ACCAGGCCGTCGAGATTGA |
| **GAPDH** | GAGTCAACGGATTTGGTCGT |
|  | TTGATTTTGGAGGGATCTCG |
| **mBcl-2** | GCTACCGTCGTGACTTCGC |
|  | CCCCACCGAACTCAAAGAAGG |
| **HPRT** | GGGGGCTATAAGTTCTTTGC |
|  | TCCAACACTTCGAGAGGTCC |
| **CD235a** | ACAACTTGCCCATCATTTCTCTG |
|  | TCAGTCGGCGAATACCGTAAG |
| **CD71** | GGCTACTTGGGCTATTGTAAAGG |
|  | CAGTTTCTCCGACAACTTTCTCT |
| **CD36** | CTTTGGCTTAATGAGACTGGGAC |
|  | GCAACAAACATCACCACACCA |
| **GATA1** | CTGTCCCCAATAGTGCTTATGG |
|  | GAATAGGCTGCTGAATTGAGGG |
| **EKLF** | GGTTGCGGCAAGAGCTACA |
|  | GTCAGAGCGCGAAAAAGCAC |
| **MYB** | GAAAGCGTCACTTGGGGAAAA |
|  | TGTTCGATTCGGGAGATAATTGG |
| **HBA** | CAACTTCAAGCTAAGCCACTGC |
|  | CGGTGCTCACAGAAGCCAG |

**Table S2. Sequence of miRNA mimics used in the article.**

| **Name** | **Sequence (5^,^ to 3^,^)** |
| --- | --- |
| **miR-125b mimics** | UCCCUGAGACCCUAACUUGUGA  ACAAGUUAGGGUCUCAGGGAUU |
| **Negative Control (NC)** | UUCUCCGAACGUGUCACGUTT  ACGUGACACGUUCGGAGAATT |
| **miR-125b Inhibitor** | UCACAAGUUAGGGUCUCAGGGA |
| **Inhibitor Negative Control (NI)** | CAGUACUUUUGUGUAGUACAA |

**Table S3. Antibodies used in in the article.**

| **Antibodies** | **Vendor** | **Catalog number** |
| --- | --- | --- |
| Anti-Human CD71-PE-Cy7 | eBioscience | cat#25-0719-42 |
| Anti-Human CD71-APC | BD bioscience | cat#551374 |
| Anti-Human CD71-FITC | BD bioscience | cat#555536 |
| Anti-Human CD235a-BV421 | BD bioscience | cat#562938 |
| Anti-Human CD235a-PE | BD bioscience | cat# 555570 |
| Anti-Human CD49d-PE | eBioscience | cat#12-0492-83 |
| LDS751 | Invitrogen | cat#L7595 |
| SYTO™ 16 Green Fluorescent Nucleic Acid Stain | eBiosciences | cat#S7578 |
| SYTO™ 62 Red Fluorescent Nucleic Acid Stain | eBiosciences | cat#S11344 |
| Anti-Mouse TER-119-PE-CY7 | eBioscience | cat#25-5921-82 |
| Anti-Mouse CD71-BV711 | BD bioscience | cat#740667 |
| Anti-Mouse CD71-PE | eBioscience | cat#12-0711-82 |
| Anti-Mouse Ter119-APC | eBioscience | cat#17-5921-82 |
| CFSE | eBioscience | cat#65-0850-84 |
| Dil | Beyotime Biotechnology | cat#C1036 |
| Actin-Stai 555 phalloidin | Cytoskeleton | cat#PHDH1 |
| Actin-Tracker Red-Rhodamine/phalloidin | Beyotime Biotechnology | cat#C2207S |
| TER-119 Monoclonal Antibody | eBioscience | cat#14-5921-82 |
| CD235a Monoclonal Antibody | Invitrogen | cat#MA1-20893 |
| Bcl-2 antibody | Santa | cat#sc-7382 |
| Bcl-2 antibody | abcam | cat#ab32124 |
| Caspase-3 Antibody | CST | cat#9662 |
| ROCK-1 Antibody | CST | cat#4035 |
| MLC2 | CST | cat#3672 |
| p-MLC2 | CST | cat#3674 |
| GAPDH Monoclonal Antibody | Earth Ox | cat#E021010 |
| HRP-conjugated GAPDH Monoclonal antibody | Proteintech | cat#HRP-60004 |
| Peroxidase AffiniPure goat anti-rabbit-IgG (H+L) | Jackson ImmunoResearch | cat#111-035-003 |

1. Tahannejad Asadi Z, Yarahmadi R, Saki N, Jalali MT, Amin Asnafi A, Tangestani R. Investigation of JAK2V617F Mutation Prevalence in Patients with Beta Thalassemia Major. *Lab Med* 2020, **51**(2)**:** 176-180.
