# Supplementary material for: The accumulation of miR-125b-5p is indispensable for efficient erythroblast enucleation
Source: Cell Death Dis. 2022 Oct 21;13(10):886. doi: 10.1038/s41419-022-05331-5 (PMC9586935; doi:10.1038/s41419-022-05331-5)
Supplement: Supplementary file 2 — Original Data File [file 41419_2022_5331_MOESM2_ESM.pptx]

## Slide 1
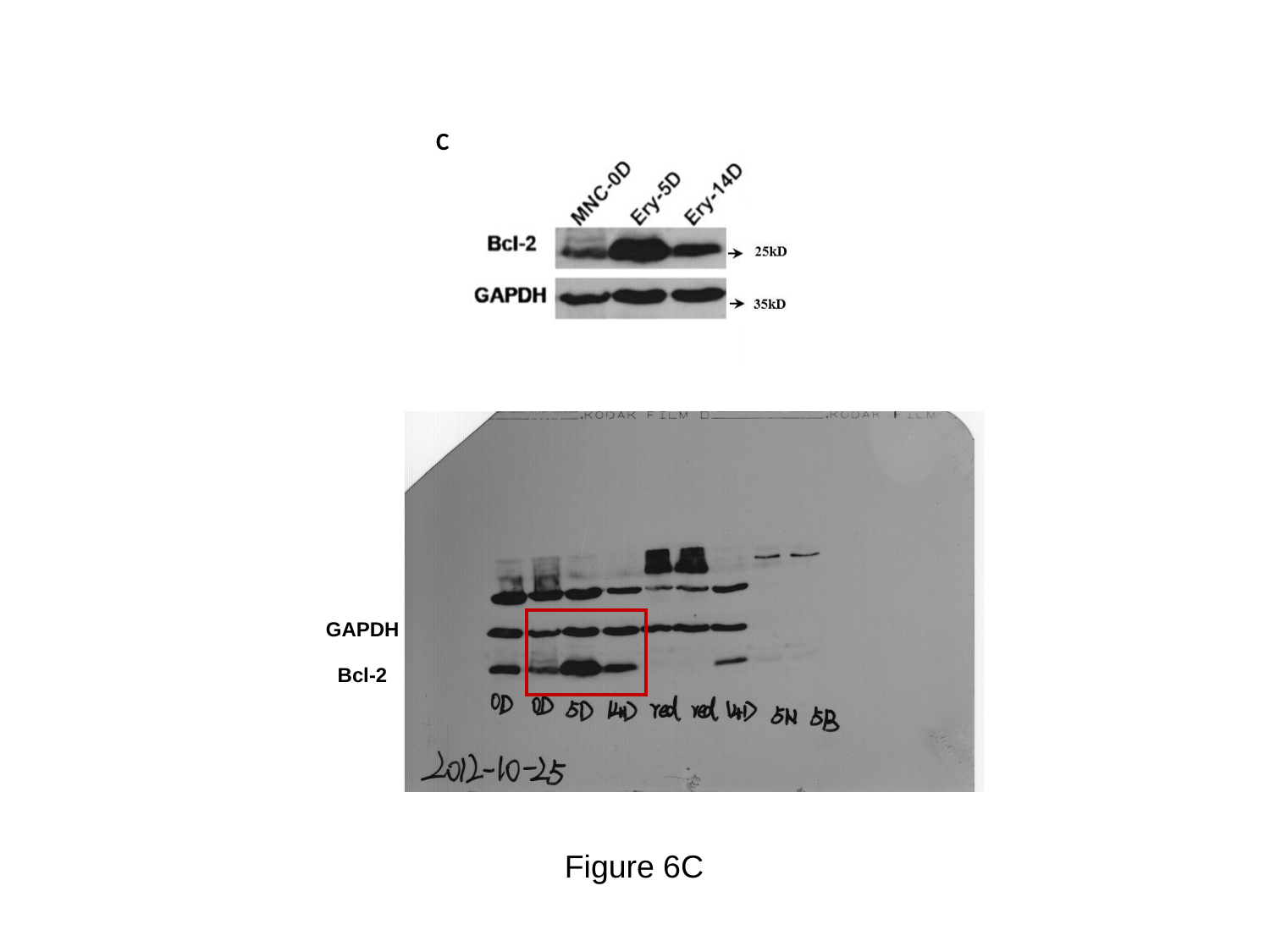

C
GAPDH
Bcl-2
Figure 6C

## Slide 2
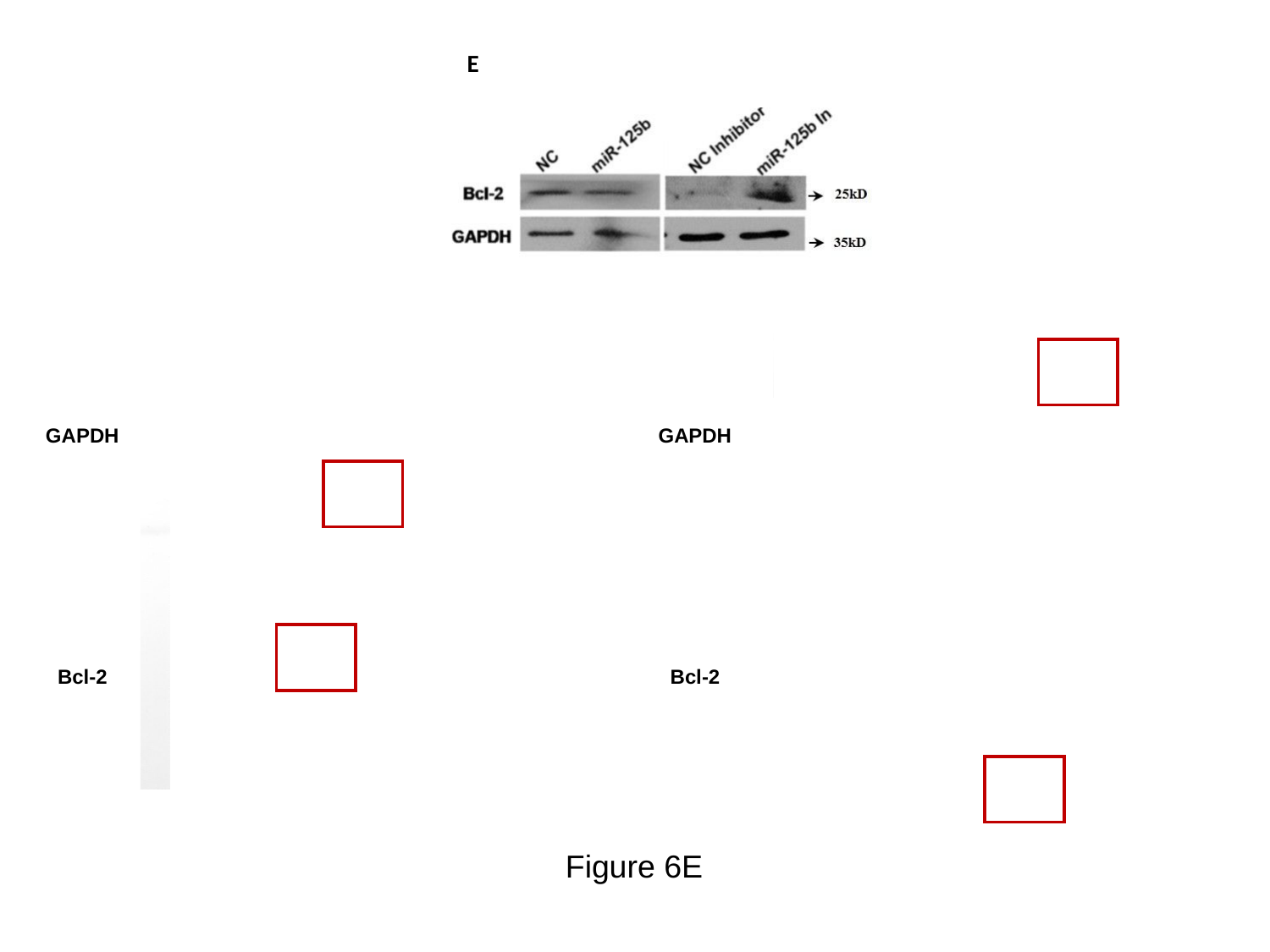

E
GAPDH
Bcl-2
GAPDH
Bcl-2
Figure 6E

## Slide 3
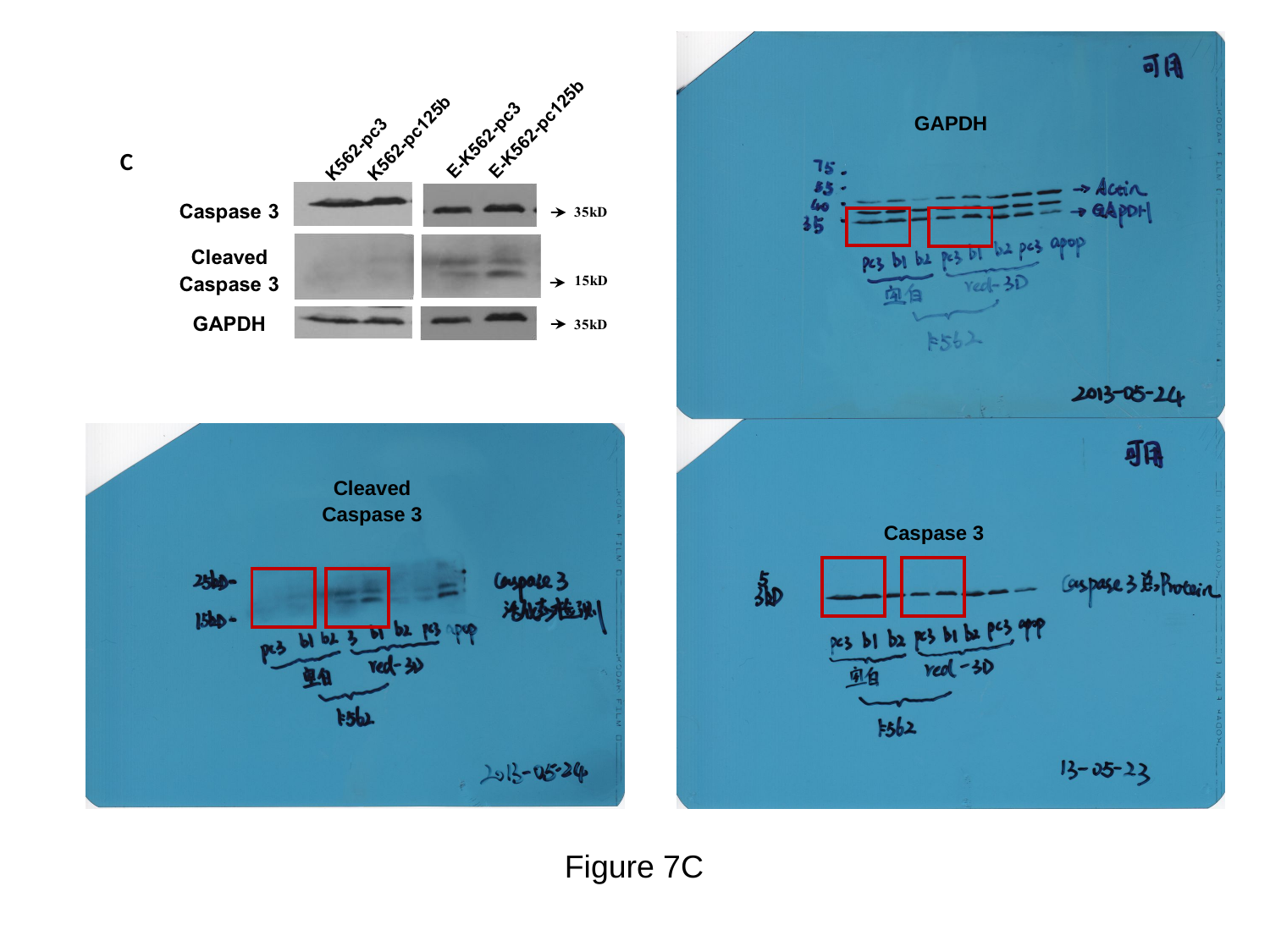

GAPDH
Caspase 3
C
Cleaved
Caspase 3
Figure 7C

## Slide 4
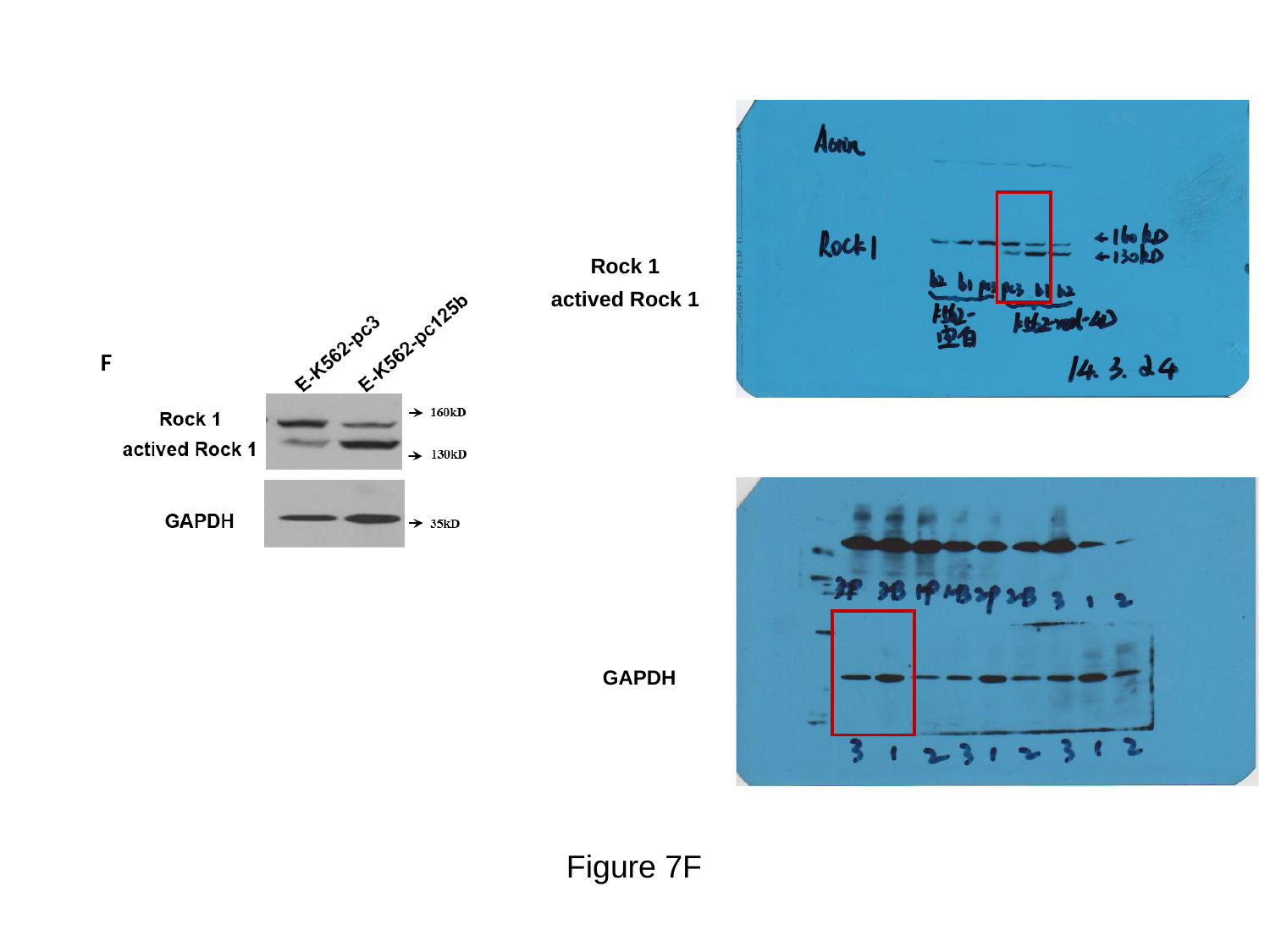

Rock 1
actived Rock 1
F
GAPDH
Figure 7F

## Slide 5
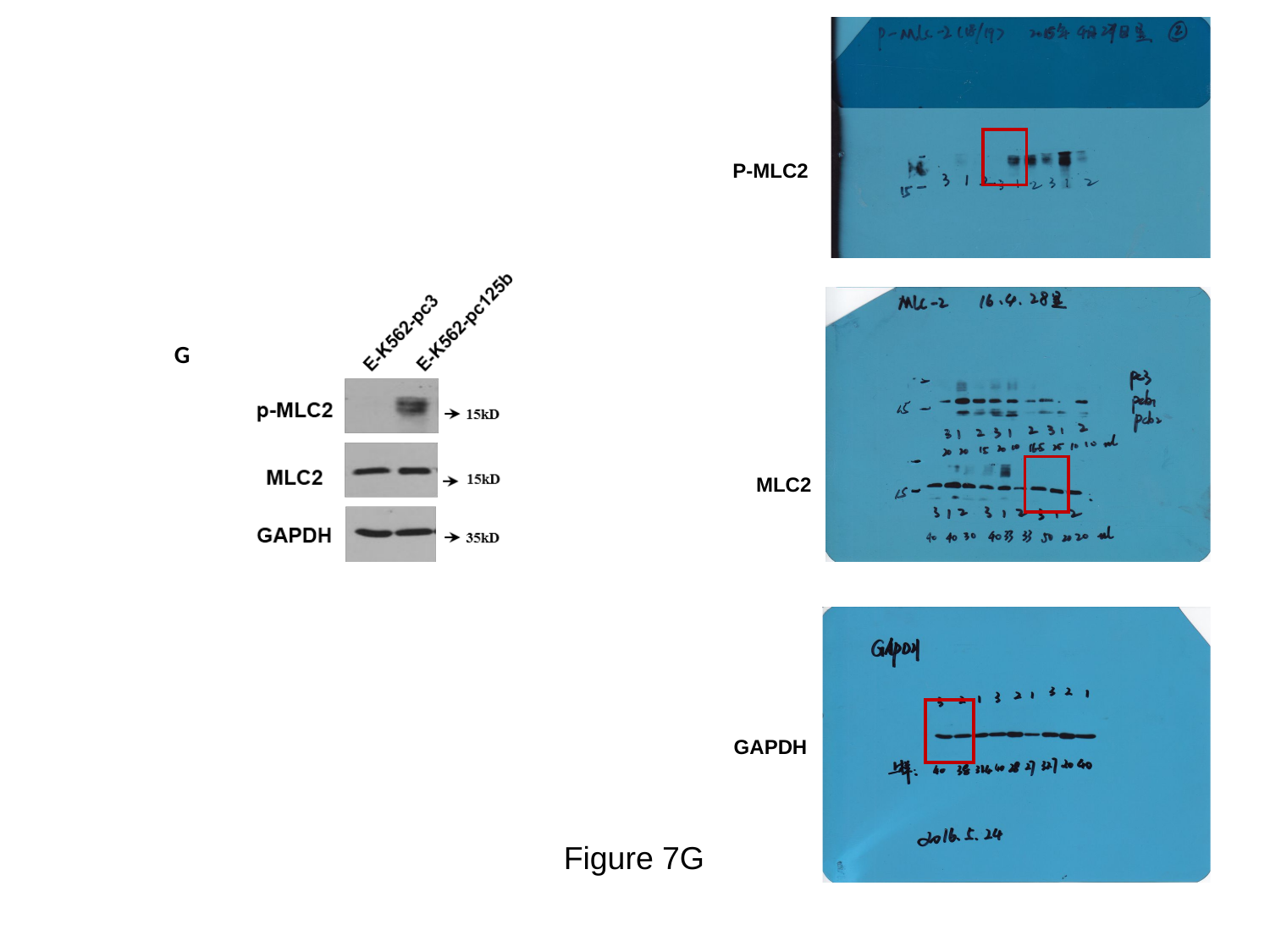

P-MLC2
MLC2
G
GAPDH
Figure 7G

## Slide 6
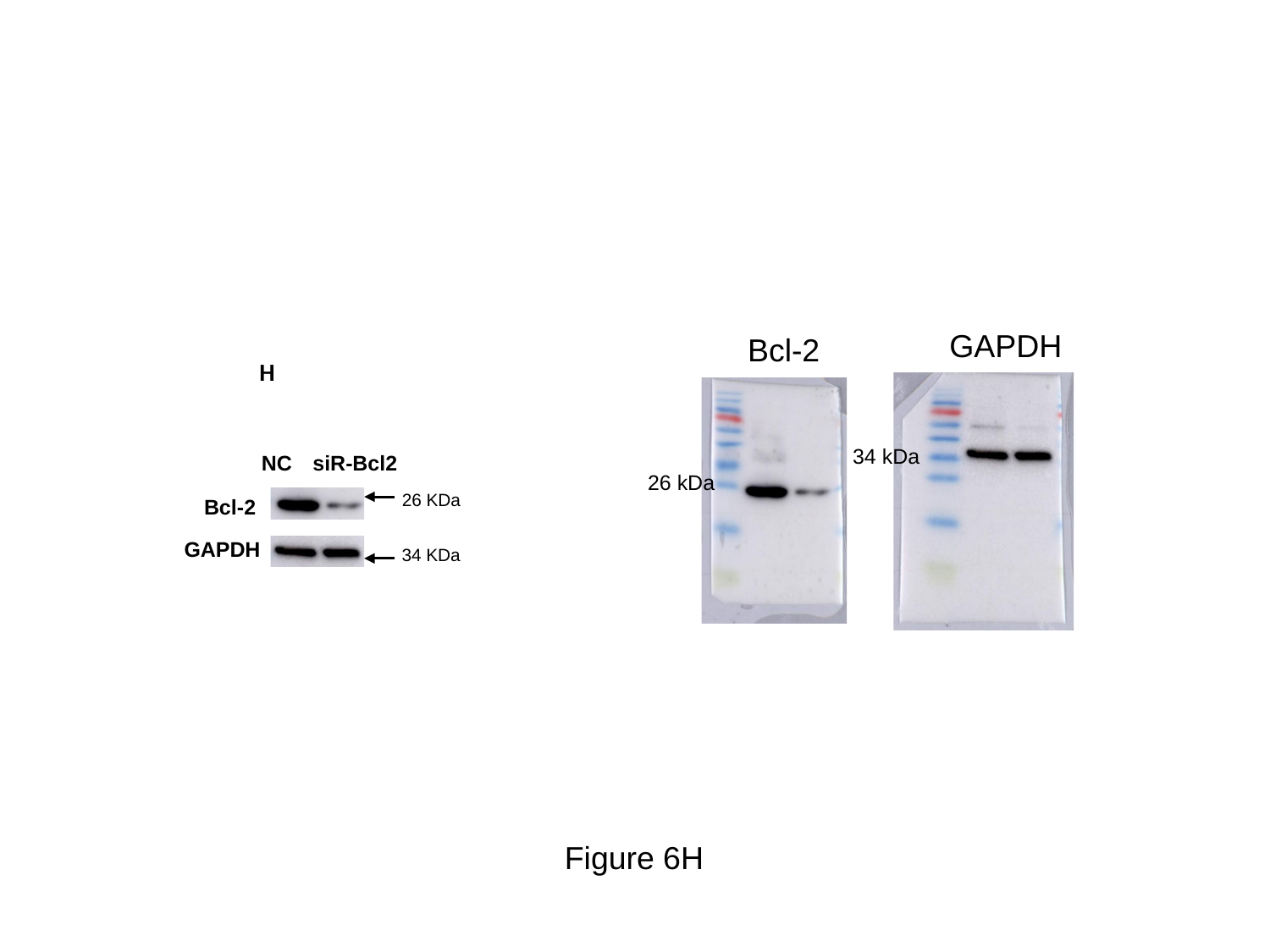

GAPDH
Bcl-2
H
34 kDa
NC
siR-Bcl2
26 KDa
Bcl-2
GAPDH
34 KDa
26 kDa
Figure 6H

## Slide 7
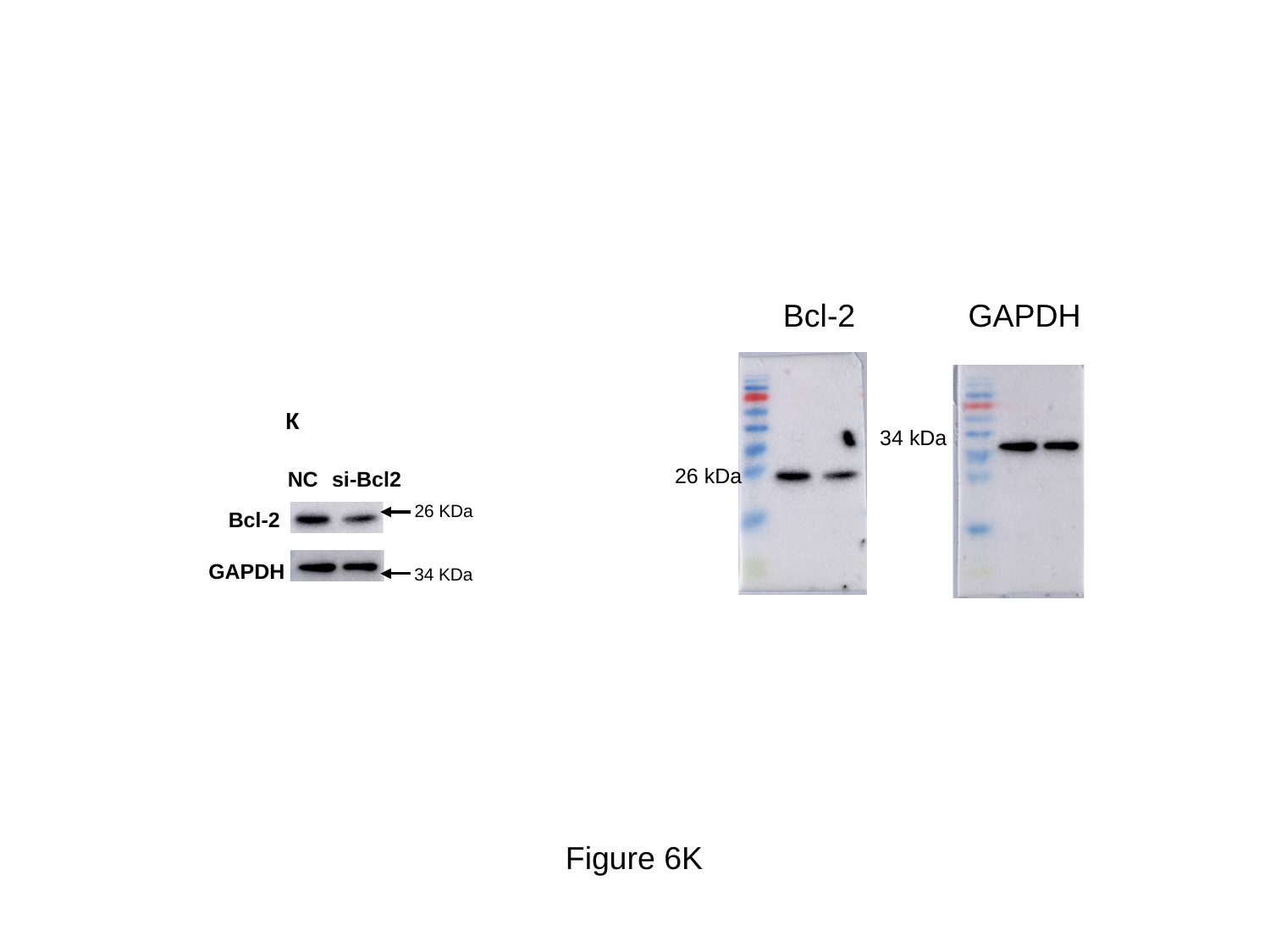

Bcl-2
GAPDH
K
34 kDa
26 kDa
NC
si-Bcl2
26 KDa
Bcl-2
GAPDH
34 KDa
Figure 6K
